# Whole exome sequencing identifies mTOR and KEAP1 as potential targets for radiosensitization of HNSCC cells refractory to EGFR and β1 integrin inhibition

**DOI:** 10.18632/oncotarget.24266

**Published:** 2018-04-06

**Authors:** Erik Klapproth, Ellen Dickreuter, Falk Zakrzewski, Michael Seifert, Andreas Petzold, Andreas Dahl, Evelin Schröck, Barbara Klink, Nils Cordes

**Affiliations:** ^1^ OncoRay – National Center for Radiation Research in Oncology, Faculty of Medicine, Technische Universität Dresden, 01307 Dresden, Germany; ^2^ German Cancer Consortium (DKTK), Dresden 01307, Germany; ^3^ German Cancer Research Center (DKFZ), Dresden partner site, Heidelberg 69120, Germany; ^4^ Core Unit for Molecular Tumor Diagnostics (CMTD), National Center for Tumor Diseases (NCT), Dresden 01307, Germany; ^5^ Institute for Medical Informatics and Biometry (IMB), Technische Universität Dresden, Dresden 01307, Germany; ^6^ National Center for Tumor Diseases (NCT), Dresden 01307, Germany; ^7^ Deep Sequencing Group, BIOTEChnology Center, Technische Universität Dresden, Dresden 01307, Germany; ^8^ Institute for Clinical Genetics, Faculty of Medicine Carl Gustav Carus, Technische Universität Dresden, Dresden 01307, Germany; ^9^ Department of Radiation Oncology, University Hospital Carl Gustav Carus, Technische Universität Dresden, Dresden 01307, Germany; ^10^ Helmholtz-Zentrum Dresden - Rossendorf, Institute of Radiooncology, Dresden 01328, Germany

**Keywords:** beta1 integrin, EGFR, Exome, ionizing radiation, HNSCC

## Abstract

Intrinsic and acquired resistances are major obstacles in cancer therapy. Genetic characterization is commonly used to identify predictive or prognostic biomarker signatures and potential cancer targets in samples from therapy-naïve patients. By far less common are such investigations to identify specific, predictive and/or prognostic gene signatures in patients or cancer cells refractory to a specific molecular-targeted intervention. This, however, might have a great value to foster the development of tailored, personalized cancer therapy. Based on our identification of a differential radiosensitization by single and combined β1 integrin (AIIB2) and EGFR (Cetuximab) targeting in more physiological, three-dimensional head and neck squamous cell carcinoma (HNSCC) cell cultures, we performed comparative whole exome sequencing, phosphoproteome analyses and RNAi knockdown screens in responder and non-responder cell lines. We found a higher rate of gene mutations with putative protein-changing characteristics in non-responders and different mutational profiles of responders and non-responders. These profiles allow stratification of HNSCC patients and identification of potential targets to address treatment resistance. Consecutively, pharmacological inhibition of mTOR and KEAP1 effectively diminished non-responder insusceptibility to β1 integrin and EGFR targeting for radiosensitization. Our data pinpoint the added value of genetic biomarker identification after selection for cancer subgroup responsiveness to targeted therapies.

## INTRODUCTION

Intrinsic and acquired resistance are major obstacles in cancer therapy [1–3]. The plethora of factors creating this resistance spans from gene mutations, over epigenetic alterations to microenvironmental cues. To achieve personalization in cancer medicine, great efforts have been undertaken to genetically characterize therapy-naïve patient tumor material to identify specific, predictive and/or prognostic biomarker signatures [4–6]. While some successes document the feasibility and translatability of these approaches, similar investigations for identifying biomarker signatures in patients and cancer cells refractory to a specific molecular-targeted intervention have been rarely accomplished [7, 8]. However, such experimental approaches might have a great added value to discriminate between responding and non-responding patients before start of treatment or uncover treatment failure early after start of treatment. The former could be made feasible by exposing tumor biopsies to the treatment of choice and monitor a particular marker or endpoint over a reasonable, clinically accountable short period of time.

The rationale for the present study is in line with this concept and based on our previously reported observation of a group of responders versus non-responders in a panel of head and neck squamous cell carcinoma (HNSCC) cell lines. In contrast to responders, the non-responders failed to show radiosensitization upon single or simultaneous administration of AIIB2 (inhibiting β1 integrin) and Cetuximab (inhibiting EGFR) [9–11]. We further showed that our *in-vitro* clonogenic cell survival analyses employing more physiological three-dimensional (3D), matrix-based HNSCC cell cultures predicted the responsiveness of HNSCC tumors grown on nude mice [9, 12]. Hence, our findings provide a unique and discriminative set of cell lines for an exome-based biomarker and target identification.

Both β1 integrin and EGFR are cell surface receptors coalescing in specific cell membrane areas called focal adhesions [13]. Mutual and cooperative interactions between these receptors allow optimized regulation of different cell functions such as survival, proliferation and migration in normal and malignant cells. Based on their overexpression and prosurvival signaling in numerous malignancies [14–17], β1 integrin and EGFR are anticipated as potential cancer targets [18, 19]. Various already clinically available and effective inhibitors can be found for EGFR. In addition to tyrosine kinase inhibitors (TKIs), Cetuximab (and other antibodies) is approved for HNSCC (together with radiotherapy; including recurrent or metastatic head and neck cancer) as well as KRAS wild-type and EGFR-expressing metastatic colorectal cancer (https://clinicaltrials.gov/). Cetuximab/radiotherapy has demonstrated significantly prolonged locoregional control and overall survival in patients with HNSCC and is appreciated as the prime example for a successful treatment composed of a molecular drug and radiotherapy. However, a comparative clinical trial with Cisplatin plus radiotherapy revealed similar potency of both approaches. Despite great advances in treating, for example HNSCC, colorectal and non-small cell lung cancer with antibodies or small molecule inhibitors, resistances to these molecular compounds have been documented. Importantly, the benefit seen with first and second generation EGFR TKIs is, in general, transient and virtually all patients become resistant [19–24].

For targeting of β1 integrins, several antibodies are available for treatment of diseases other than cancer [25–27]. For human malignancies, very few anti-integrin agents (i.e. antibodies, peptidomimetics) have been evaluated, which mainly block αV and α5 integrin [28–33]. Clinical trials evaluating the efficacy of anti-integrin agents as monotherapy or together with radiochemotherapy only found a minor or no curative potential. With regards to the limited inhibitory spectrum of these compounds and the generally accepted fact that integrin-mediated adhesion fundamentally impacts on cancer cell therapy resistance, we and others have focused on β1 integrin targeting to deactivated, most favorable, all 12 integrin receptors β1 integrin is involved in. While no clinical trials are currently underway together with radiotherapy, OS2966, a humanized derivative of AIIB2, has been evaluated highly successful in preclinical studies and its clinical testing is anticipated. The idea to therapeutically inhibit integrins in the clinic arises from own reports and reports from others that integrin targeting is a potent approach to enhance cancer cell radiochemosensitivity in various preclinical cancer models [9, 12, 34–37]. Upon detailed characterization of our HNSCC three-dimensional (3D) cell culture and animal models, a reciprocal prosurvival bypass signaling induced by β1 integrin (AIIB2) or EGFR targeting (Cetuximab) led us to a simultaneous targeting of both receptors for radiosensitization [12]. Interestingly, the HNSCC cell line panel remained to be grouped into responders and non-responders under AIIB2/Cetuximab comparable to AIIB2 or Cetuximab monotherapies.

Here, we used HNSCC as model to further define genetic differences in responders versus non-responders to a combination of a clinically approved and an experimental drug by performing comparative whole exome sequencing and phosphoproteome analyses in cell lines grown under more physiological 3D conditions. We found a higher rate of gene mutations with putative protein-changing characteristics in non-responders, mutational profiles of responders and non-responders that allow stratification of HNSCC patients, and pharmacological inhibition of Kelch Like ECH Associated Protein 1 (KEAP1) and mechanistic target of rapamycin (mTOR) to effectively diminish non-responder insusceptibility to β1 integrin and EGFR targeting for radiosensitization. Our data pinpoint the added value of genetic biomarker identification after selecting for cancer subgroup responsiveness to targeted therapies.

## RESULTS

### Comparative whole exome analysis of responder and non-responder HNSCC cells

**Figure 1:**
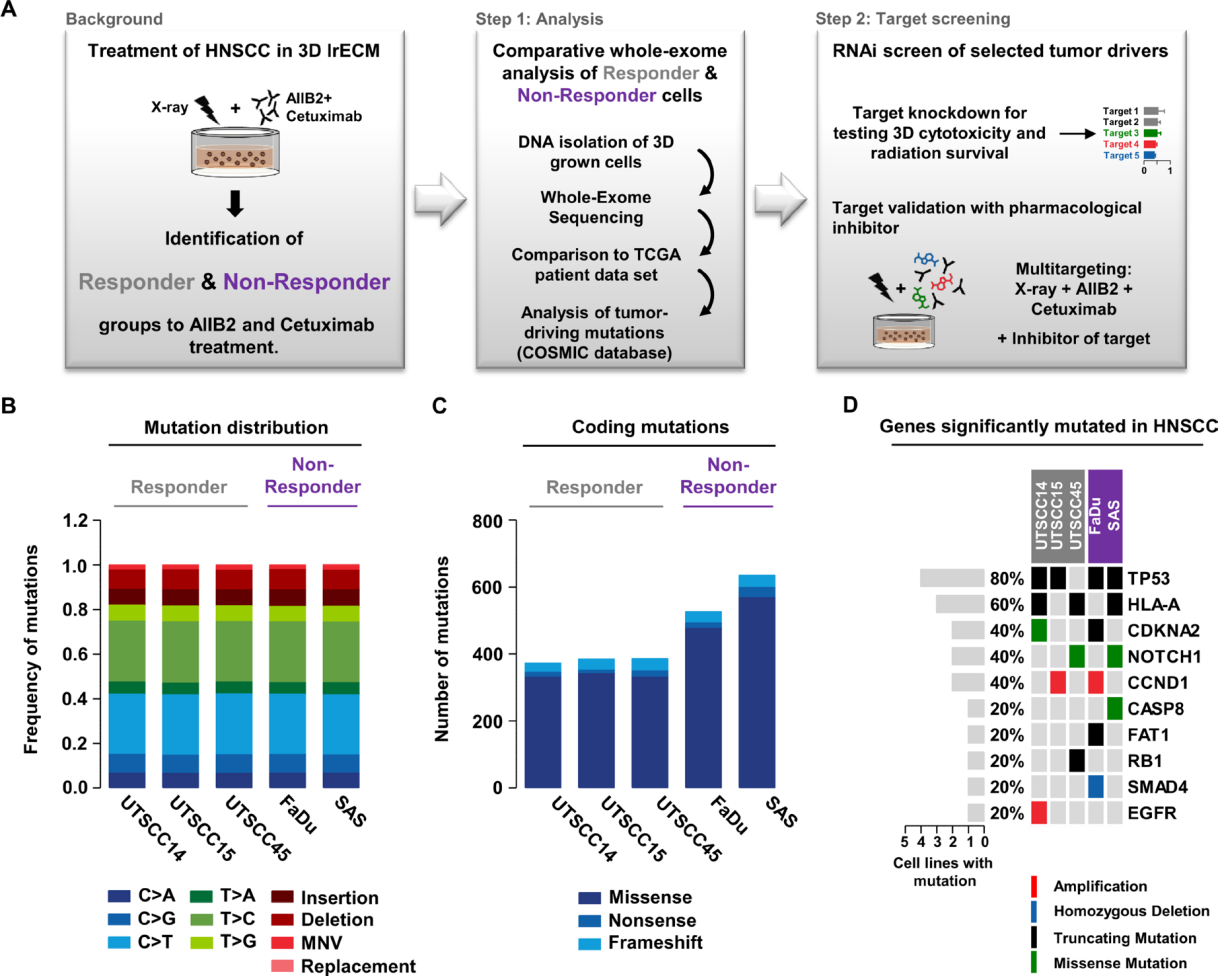
Comparative whole exome sequencing analysis of responder and non-responder HNSCC cells (**A**) Background and workflow of experimental setups. (**B**) Distribution of single-nucleotide variants, insertions, deletions, multi-nucleotide variants (MNV) and replacements detected in the tested responder (grey) and non-responder (purple) 3D grown HNSCC cell lines. (**C**) Distribution of coding mutations (missense, nonsense and frameshift mutations) measured in tested HNSCC cell lines. (**D**) Matrix of genes most frequently mutated in HNSCC patients and their mutation appearance in 3D lrECM grown responder (grey) and non-responder (purple) cell lines.

Based on our rationale and methodological set-up (Figure 1A), we performed whole exome sequencing on both treatment-naïve responder (susceptible to AIIB2/Cetuximab-driven radiosensitization; UTSCC14, UTSCC15, UTSCC45) and non-responder (insusceptible to AIIB2/Cetuximab-driven radiosensitization; FaDu, SAS) HNSCC cell lines. In total, we found 160,00 to 205,000 genomic variants (Supplementary Figure 1 and Supplementary Table 1) with a very similar frequency of base exchanges, insertions, deletions, multiple nucleotide variations (MNV) and replacements in responders and non-responders (Figure 1B and Supplementary Figure 2). Analysis of protein-coding variants revealed higher numbers in non-responders relative to responders with regard to missense, nonsense, and frameshift mutations (Figure 1C). Then, we matched the gene mutation frequency, in terms of amplification, homozygous deletion, truncating mutations, and missense mutations, of major tumor drivers in HNSCC with mutated genes from our cell line panel. We found a similar distribution with, for example, mutated TP53 in 4 out of 5 cell lines (71% mutated in HNSCC patients) and mutated CDKNA2 in two out of five cell lines (22% mutated, 60% gene copy loss in HNSCC patients) (Figure 1D) [38].

### Mutational profiles of responder and non-responder cells allow stratification of HNSCC patients

**Figure 2:**
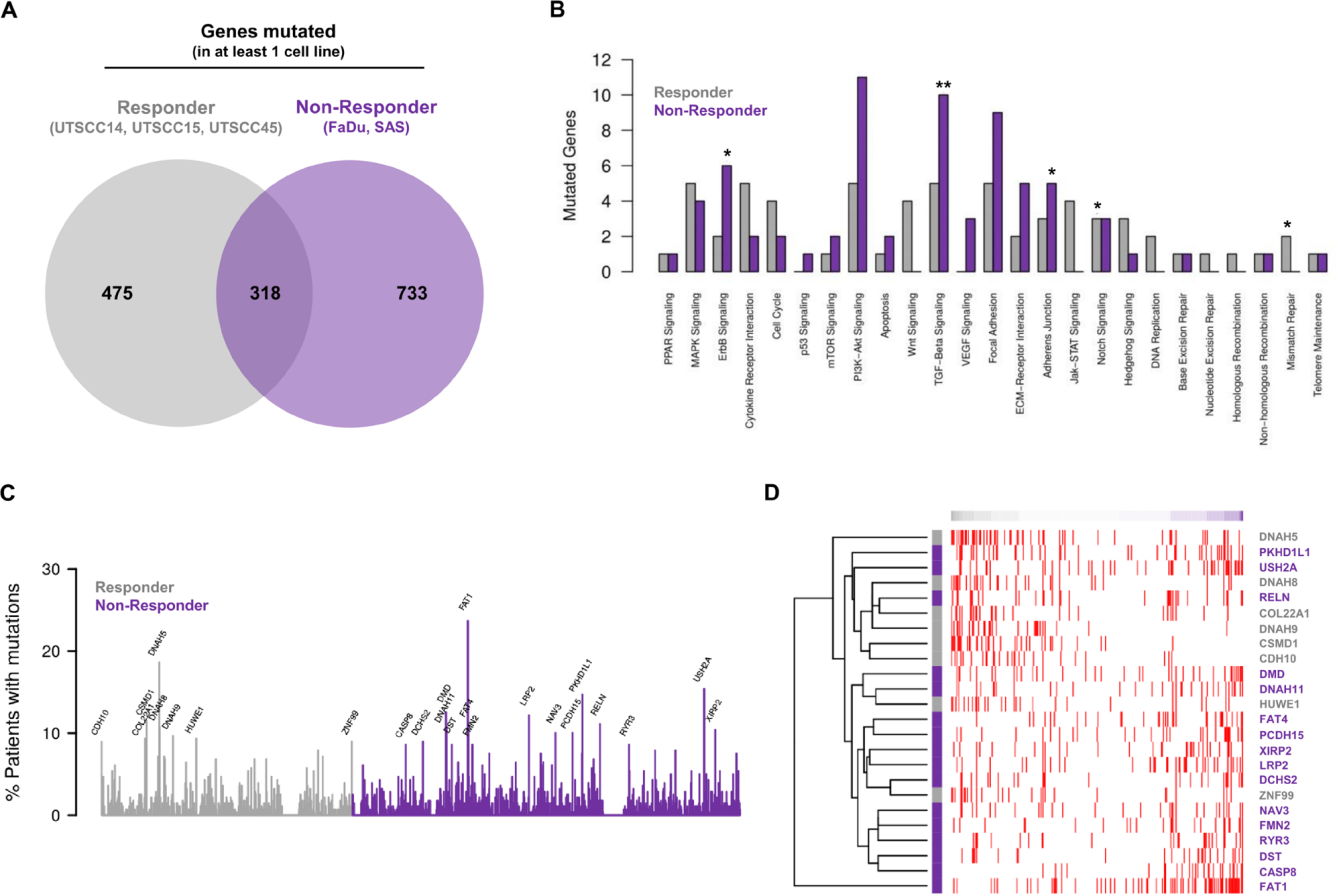
Mutational profiles of responder and non-responder cells allow stratification of HNSCC patients (**A**) Venn diagram analysis of recurrently mutated genes (found to be mutated in at least one out of five cell lines) showing shared and exclusively mutated genes in the responder or non-responder groups. (**B**) Analysis of cancer associated signaling pathways affected by exclusively mutated genes found for responder and non-responder cell lines. Numbers of mutated genes in each known cancer-relevant signaling pathway are shown. Significant enrichment of mutated genes in a pathway of a group is highlighted by ^*^ for *P* < 0.1 and by ^**^ for *P* < 0.05 (Fisher's exact test). (**C**) Analysis of mutation frequencies of exclusively mutated genes found for responder and non-responder cell lines within the HNSCC cohort of the TCGA. Only names of genes mutated in at least 8% of the patients are shown. (**D**) Exclusively mutated genes found for responder and non-responder cell lines allow determination of the similarity of TCGA HNSCC patients with potential responder and non-responder profiles. Patient-specific gene mutation profiles were compared with the corresponding characteristic profile of the responder and non-responder cells considering genes mutated in at least 8% of the TCGA HNSCC cohort (C). Heatmap shows individual mutation profiles of patients (columns) for the selected genes (rows). Gene mutations are highlighted in red in the heatmap. Patients are sorted according to the similarity of their gene mutation profiles from potential responder (left) to non-responder profiles (right). Colors of the gene names on the right side of the heatmap represent their exclusive presence in either responder (grey) or non-responder cell lines (purple).

Next, we employed genes found to have mutations likely to affect protein function for comparative analysis of exclusive mutations from responders and non-responders. Here, 475 mutated genes were unique for responders, 733 for non-responders and 318 overlapped (Figure 2A and Supplementary Figure 3A).

To explore the signaling pathways these genes are involved in, we mapped the genes exclusively mutated in at least one responder or in at least one non-responder cell line to known cancer signaling pathways. As shown in Figure 2B, the mutated genes allocated to numerous signaling pathways involved in cancer progression and therapy resistance. Significant gene enrichment in responders was found for Notch and mismatch repair signaling and in non-responders for ErbB, TGF-β and adherens junction signaling (Figure 2B).

Subsequently, the mutation frequency of curated genes exclusively mutated in at least one responder or non-responder cell line was uncovered in the HNSCC cohort of the TCGA (*n* = 279). Our analysis of genes mutated in at least 8% of HNSCC patients interestingly showed the presence of these mutated genes in the TCGA HNSCC patient cohort to a varying extent (Figure 2C). Further, the exclusively mutated genes found in responder and non-responder cell lines allowed determination of similarity between TCGA HNSCC patients and potential responder and non-responder profiles (Figure 2D).

### Cytotoxicity and radiosensitization upon knockdown of a selected set of mutated genes in HNSCC non-responder cell lines

**Figure 3:**
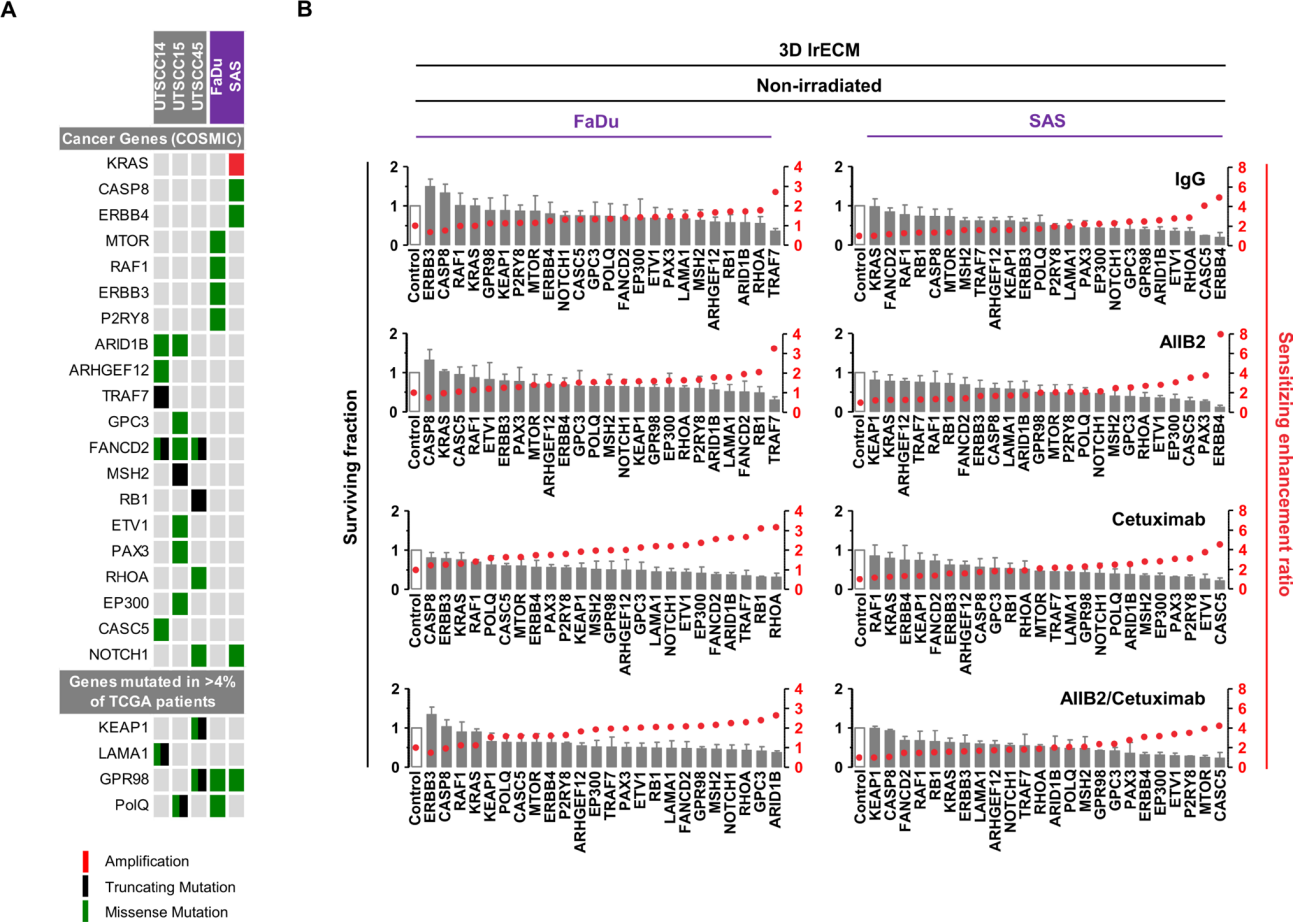
Cytotoxicity in FaDu and SAS non-responders cell lines upon esiRNA-mediated knockdown of tumor-driving genes (**A**) Matrix showing tumor drivers mutated in responder and non-responder cells. Selection of candidates was based on the type of mutation, available information on gene function and association with cancer or therapy response (e.g. genes included in the COSMIC CENSUS list; http://cancer.sanger.ac.uk/census/; mutated in >4% of cancers based on the TCGA dataset). (**B**) Cytotoxicity measured as clonogenic survival of FaDu and SAS non-responder cells upon esiRNA-mediated knockdown of the 24 indicated genes identified by whole exome sequencing. In addition to knockdown, cells were treated with AIIB2 and Cetuximab alone or in combination (RLUC esiRNA and IgG were used as control). Surviving fraction (grey bars) and sensitizing enhancement ratio (red dots) upon indicated treatments are presented. Results show mean ± SEM, *n* = 3, two-sided *t*-test, ^*^P < 0.05; ^**^P < 0.01; ^***^P < 0.001.

To identify novel potential cancer targets whose inhibition is likely to effectively diminish non-responder insusceptibility to β1 integrin and EGFR targeting for radiosensitization, we applied a more restrictive filtering to uncover genes mutated in at least two cell lines. Four-hundred and one mutated genes were discovered of which 47 were unique for responders (2 out of 3), 29 for non-responders (2 out of 2) and 325 overlapped between the responder and non-responder group (Supplementary Figure 3A and 3B). Results of this analysis were combined with the analysis of the type of mutation, their status as cancer-relevant genes (based on the COSMIC CENSUS list at http://cancer.sanger.ac.uk/census/), the frequency of mutation in the TCGA cohort, and whether the various mutations supposedly result in loss or gain of function in a particular gene based on their type of mutation detected by our whole exome sequencing (Figure 3A; also see Figure 1, Supplementary Figure 3A and 3B). Conclusively, we defined a list of 24 genes (Figure 3A and Supplementary Table 2).

We then determined the cytotoxicity in 3D lrECM cell cultures of AIIB2, Cetuximab or AIIB2/Cetuximab-treated non-responders FaDu and SAS upon esiRNA-mediated silencing of these 24 genes. The knockdown had a differential, partially significant impact on cell survival with sensitizing enhancement ratios ranging from 0.5 to 8 relative to controls (Figure 3B and Supplementary Tables 3 and 4).

**Figure 4:**
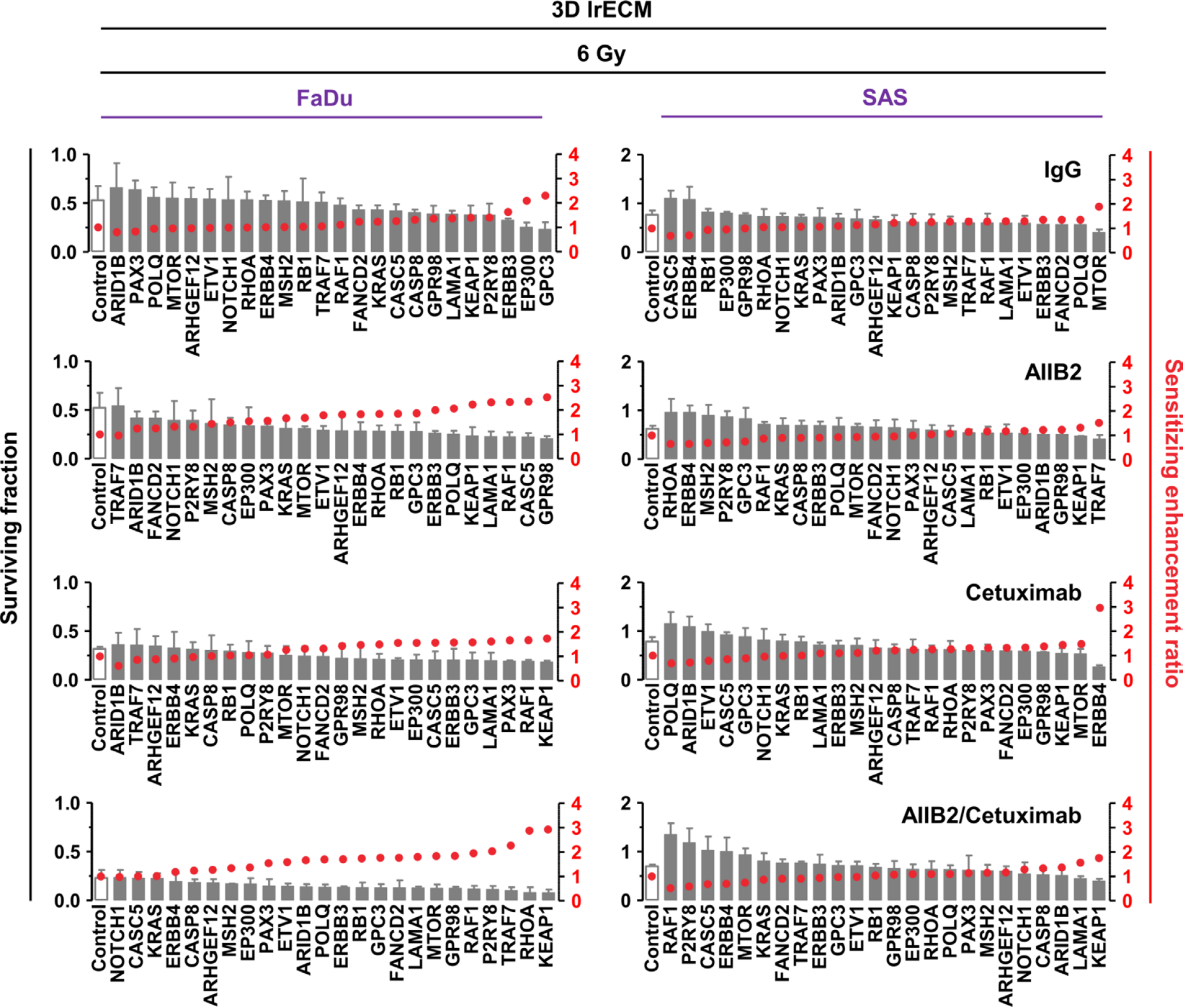
Differential radiosensitization upon esiRNA mediated knockdown of tumor-driving genes mutated in FaDu and SAS non-responder cell lines Clonogenic radiation survival (6 Gy) of the non-responder cell lines FaDu and SAS upon esiRNA-mediated knockdown of 24 indicated genes identified by whole exome sequencing. In addition to knockdown, cells were treated with AIIB2 and Cetuximab alone or in combination (RLUC esiRNA and IgG were used as control). Surviving fraction (grey bars) and sensitizing enhancement ratio (red dots) upon indicated treatments are presented. Results show mean ± SEM, *n* = 3, two-sided *t*-test, ^*^P < 0.05; ^**^P < 0.01; ^***^P < 0.001.

**Figure 5:**
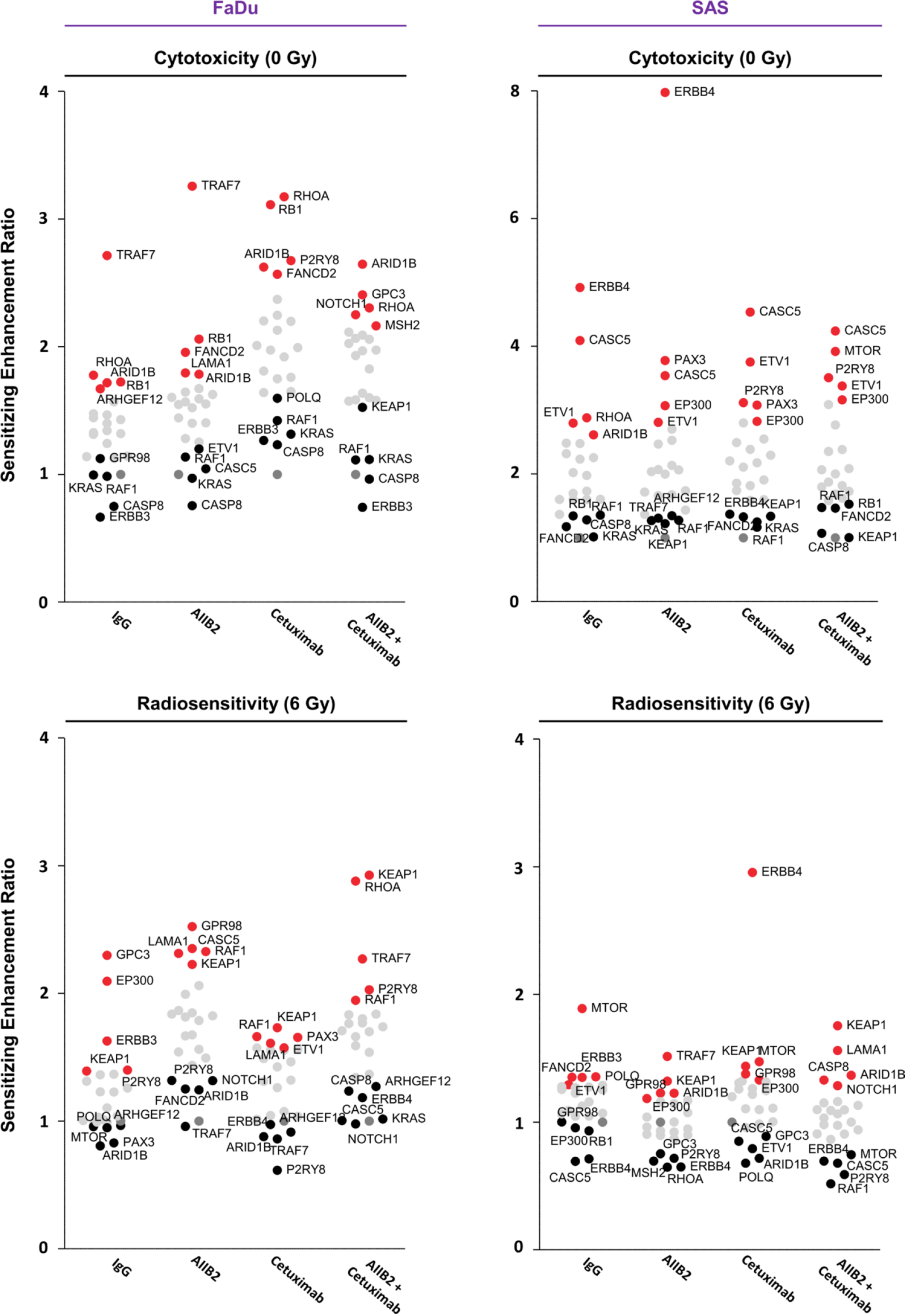
Summary of the results of the esiRNA screen Dot plot summarizing the effects of esiRNA-mediated knockdown on cytotoxicity and radiosensitivity of FaDu and SAS non-responder cells. In each row, the top 5 genes whose knockdown modifies either most ineffectively (black) or most effectively (red) the cytotoxicity or radiosensitivity of FaDu and SAS cells are presented. Knockdown was combined with IgG, AIIB2, Cetuximab or AIIB2/Cetuximab as indicated.

By combining gene silencing with AIIB2, Cetuximab or AIIB2/Cetuximab and radiotherapy, sensitizing enhancement ratios ranged from 0.5 to 3 (Figure 4 and Figure 5 and Supplementary Tables 3 and 4). Thus, the spectrum of ineffective and effective approaches found with regards to radiosensitization was different from the spectrum observed for cytotoxicity. The top 5 ineffective and top 5 effective targeting approaches in 3D lrECM FaDu and SAS non-responder cell cultures showed some similarity over all treatment groups (Figures 4 and 5).

**Figure 6:**
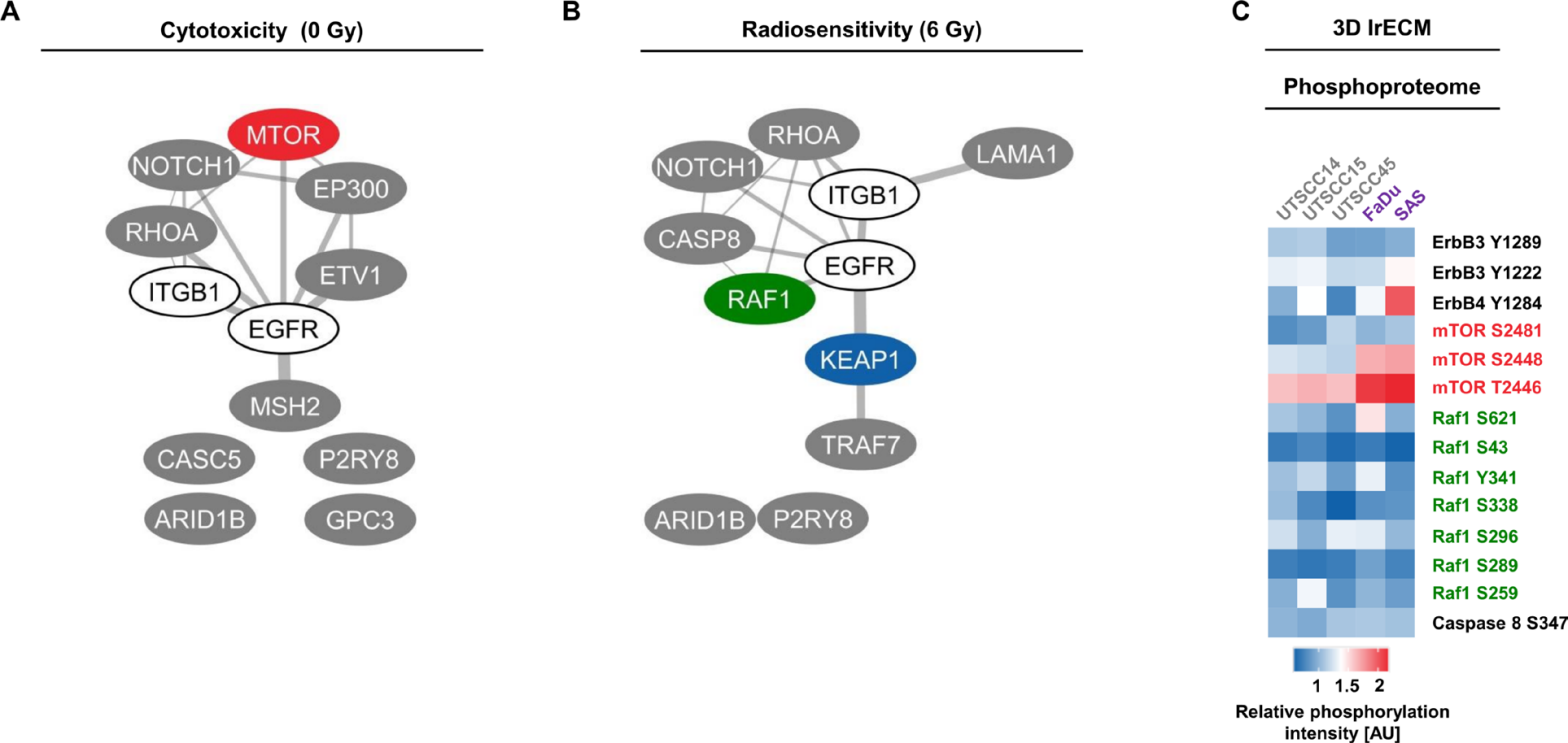
Analysis of top 5 cytotoxic and radiosensitizing candidates identified through esiRNA screening (**A and B**) Interactome of proteins whose targeting resulted in (A) cytotoxicity and (B) radiosensitization of FaDu and SAS non-responder cells upon AIIB2/Cetuximab (calculated by Cytoscape). (**C**) Heatmap shows phosphorylation intensities of selected proteins in responder and non-responder cell lines determined by broad-spectrum phosphoproteome analysis (606 phospho-specific antibodies from over 30 signaling pathways (Full Moon Biosystems); full data set see Supplementary Table 5). AU, arbitrary units.

In Figure 5, we provide a summary of overlapping genes which either most ineffectively or most effectively modified FaDu and SAS cytotoxicity and radiosensitivity. Interestingly, depletion of ERBB3, CASP8, RAF1 and KRAS seem to have no or only minor impact on FaDu cell survival in contrast to depletion of ARID1B, RB1, RHOA independent from AIIB2 or Cetuximab (Figure 5). Upon irradiation of FaDu cells, ARID1B knockdown was without effects and KEAP1 or RAF1 silencing enhanced cellular radiosensitivity irrespective of AIIB2 or Cetuximab treatment (Figure 5). In AIIB2 and Cetuximab-treated SAS cells, we found KEAP1, KRAS, RAF1 or FANCD2 knockdown without impact on cytotoxicity and EP300, ETV1 and CASC5 depletion to enhance the cytotoxicity (Figure 5). In contrast to CASC5 and ERBB4 silencing, knockdown of MTOR and KEAP1 led to radiosensitization unassociated with AIIB2 and Cetuximab (Figure 5). Further, we performed a bioinformatic Cytoscape analysis to visualize prominent interactions between the top 5 identified genes whose knockdown elicits either enhanced cytotoxicity (Figure 6A) or radiosensitivity (Figure 6B) in FaDu and SAS non-responder cells upon AIIB2/Cetuximab.

In addition to whole exome sequencing, a broad spectrum phosphoproteome investigation on whole cell lysates from untreated responder and non-responder 3D lrECM cell cultures was performed. As the phosphoproteome indicates the activity/inhibition state of a protein, we matched the protein phosphorylation status with our exome data to increase the probability to identify key prosurvival signaling mediators. Among the genes fundamental to cytotoxicity and radiosensitivity, merely mTOR showed elevated phosphorylation at serine 2481, serine 2448 and threonine 2446 in FaDu and SAS non-responders compared to UTSCC14, UTSCC15 and UTSCC45 responders (Figure 6C and Supplementary Table 5).

### Pharmacological targeting of mTOR and KEAP1 overcomes insusceptibility to AIIB2/Cetuximab-mediated radiosensitization of FaDu and SAS non-responder cells

**Figure 7:**
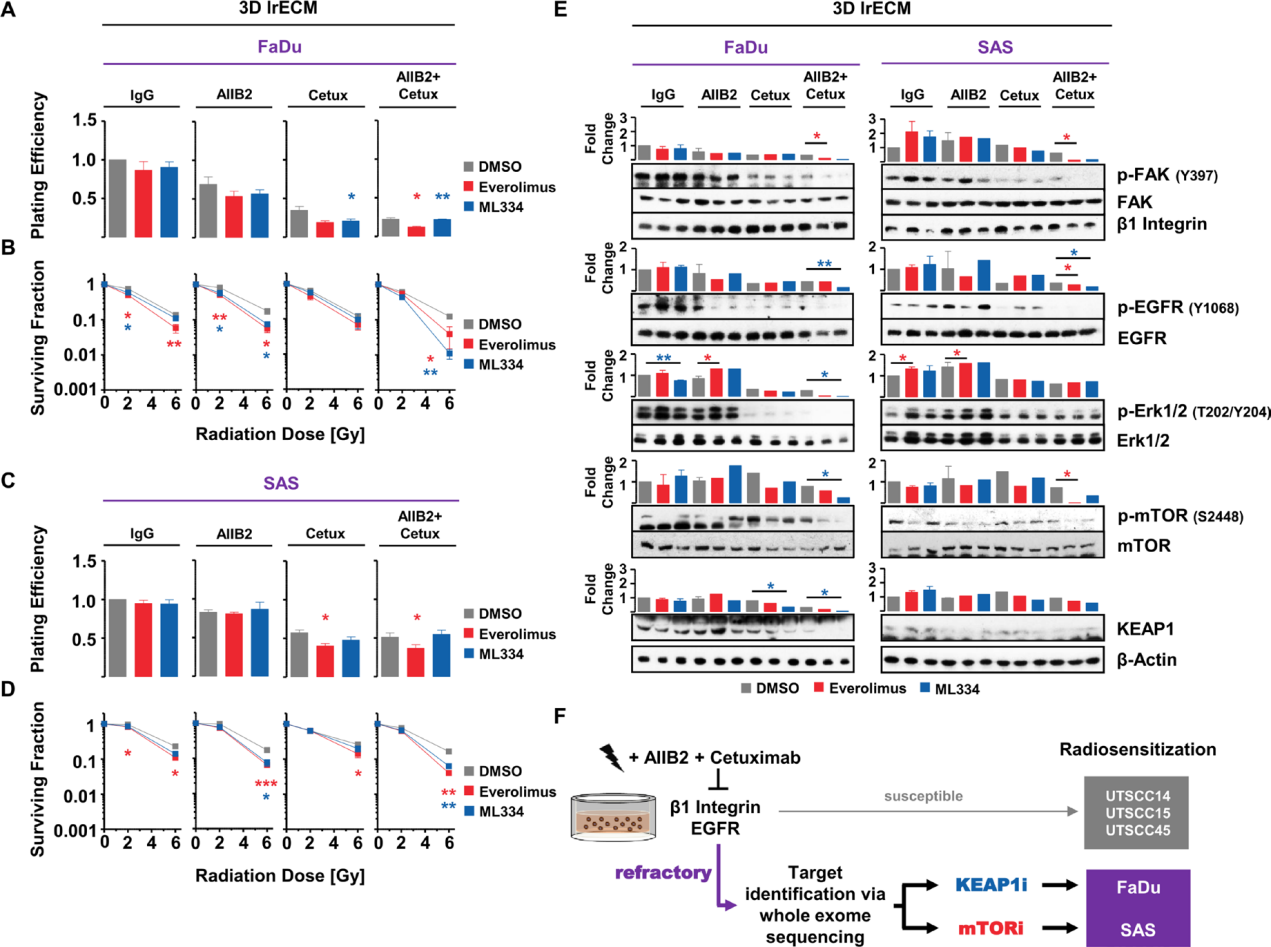
Pharmacological inhibition of KEAP1 and mTOR overcomes insusceptibility of FaDu and SAS non-responder cells to AIIB2/Cetuximab-mediated radiosensitization (**A**) Cytotoxicity measured as clonogenic survival and (**B**) clonogenic radiation survival of FaDu cells upon treatment with 10 nM Everolimus (mTOR inhibitor; EC10) or 4.5 μM ML334 (KEAP1i; EC10) simultaneously to AIIB2, Cetuximab or AIIB2/Cetuximab plus 2–6 Gy X-rays (DMSO, IgG and 0 Gy as control). (**C**) Cytotoxicity measured as clonogenic survival and (**D**) clonogenic radiation survival of SAS cells upon treatment with 65 nM Everolimus (mTOR inhibitor; EC10) or 3.3 μM ML334 (KEAP1i; EC10) simultaneously to AIIB2, Cetuximab or AIIB2/Cetuximab plus 2–6 Gy X-rays (DMSO, IgG and 0 Gy as control). (**E**) Western blots and densitometry on whole cell lysates of FaDu and SAS 3D lrECM cell cultures upon indicated treatment. Phosphorylation changes are shown for the corresponding target/readout proteins for the signaling pathways affected by β1 integrin, EGFR, mTOR and KEAP1 inhibition (DMSO and IgG as control). (**F**) Schematic depiction how non-responder cells originally refractory to AIIB2/Cetuximab treatment can be characterized by whole exome sequencing and rendered more radiosensitive by combining a KEAP1 or mTOR inhibitor to the double AIIB2/Cetuximab targeting. Results show mean ± SEM, *n* = 3, two-sided *t*-test, ^*^P < 0.05; ^**^P < 0.01.

Amongst the shared top 5 gene candidates enabling enhanced radiosensitivity upon AIIB2 and Cetuximab, KEAP1 was the most prominent hit from the esiRNA screen. mTOR was identified as potential target from our phosphoproteome analysis due to its elevated phosphorylation status in non-responder cells. Conclusively, we focused on both proteins for further analysis and deactivated mTOR and KEAP1 with an already clinically applied (Everolimus) and an experimental compound (ML334; due to lack of clinically approved KEAP1-inhibiting drugs), respectively. Based on the determination of the 10% effective concentrations of Everolimus and ML334 (Supplementary Figure 4A and 4B), we tested the cytotoxic effects of Everolimus and ML334 in combination with AIIB2, Cetuximab and irradiation. In 3D lrECM FaDu and SAS non-responder cell cultures, Everolimus resulted in differential, cell-line-dependent cytotoxicity with significant reduction of clonogenic cell survival when combined with AIIB2/Cetuximab in FaDu cells and with Cetuximab and AIIB2/Cetuximab in SAS cells relative to DMSO controls (Figure 7A and 7C). While SAS cells failed to show higher cytotoxicity upon ML344, FaDu cell survival was significantly diminished in combination with Cetuximab and AIIB2/Cetuximab (Figure 7A and 7C).

In combination with irradiation, both Everolimus and ML334 significantly enhanced radiosensitivity in FaDu and SAS cell cultures particularly when given simultaneously with AIIB2 or AIIB2/Cetuximab (Figure 7B and D). Obviously, the combination of Cetuximab plus either compound failed to achieve a strong radiosensitizing effect in the two non-responder cell lines. To note, the cytotoxic and radiosensitizing effects reached with the two pharmacological inhibitors, i.e. Everolimus and ML334, are higher relative to knockdown. Expectedly, effects differ between these approaches due to various molecular reasons such as protein presence and off-target effects. Underpinning the selectivity for non-responders of our novel identified targets, no additional or significant radiosensitization was achieved neither by Everolimus nor by ML334 in the responder cell lines UTSCC15 and UTSCC45 (Supplementary Figure 5A–5D).

Analysis of either the target molecule itself or a readout protein downstream of the target molecule revealed the potency of the combination therapies for deactivating the corresponding signaling pathway (Figure 7E). In line with previously published data [12], AIIB2 was unable to lead to a dephosphorylation of FAK. Intriguingly, Everolimus and KEAP1 application on top of AIIB2/Cetuximab either completely abrogated or decreased the phosphorylation of FAK, EGFR, Erk1/2 and mTOR (Figure 7E). These data indicate the potential to deactivate β1 integrin and EGFR associated prosurvival and promitotic signaling through mTOR and KEAP1 targeting in HNSCC cells refractory to β1 integrin and EGFR targeting.

## DISCUSSION

Therapy resistant tumors or tumor cell subpopulations greatly challenge current cancer therapies. Accordingly, there are intensive efforts on the genetic, epigenetic and proteomic level to identify predictive or prognostic biomarker signatures and potential cancer targets [39, 40]. Here, we used HNSCC cells refractory to β1 integrin/EGFR inhibition-mediated radiosensitization as model to discover mutational profiles for novel target identification. In the present study, we show (i) a higher rate of gene mutations with putative protein-changing characteristics in non-responders to β1 integrin and EGFR targeting, (ii) mutational profiles of responders and non-responders that allow stratification of HNSCC patients, and (iii) pharmacological inhibition of KEAP1 and mTOR to effectively diminish non-responder insusceptibility to β1 integrin and EGFR targeting for radiosensitization.

In contrast to the wealth of studies reporting the “effective” mechanisms underlying cytotoxicity and radiochemosensitization, studies exploring the intrinsic and acquired mechanisms of cancer cells refractory to a particular molecular therapy are scarce. It seems, however, that the non-responding tumors and tumors with early on-set of resistance require much more of our attention. Current molecular-to-macroscopic diagnostic practice does not allow broad spectrum detection and discrimination between tumors that are refractory to therapy in total or large parts and those with only a small number of resistant subpopulations. Moreover, ethical limits forbid multiple biopsies from solid tumors during therapy. Hence, one can anticipate two action paths: Liquid biopsies for multiple, time-independent observations of blood biomarkers. Although they are frequently accomplished in clinical trials, caveats are our current spectrum of reasonable biomarkers and lack of functional knowledge. A second approach is the use of tumor biopsy material in predictive assays from therapy-naïve patients. When culturing such biopsies under more physiological growth conditions such as 3D matrix, testing of responsiveness to a panel of compounds is feasible. Caveats for the latter are biopsy size and identification of clinically relevant molecular endpoints to achieve a result in a clinically justifiable time period.

In the presented work, we investigated the gene mutation profiles of HNSCC cells grown in a more physiological, ECM-based environment. The used cell lines can be either regarded as sensitive or insensitive tumors or as sensitive or insensitive subpopulations of the same tumor. Nonetheless, three cell lines (i.e. UTSCC14, UTSCC15, UTSCC45) are susceptible to AIIB2/Cetuximab-mediated radiosensitization while two (i.e. FaDu, SAS) are not. Our comparative whole exome analysis of responder and non-responder HNSCC cells revealed similarity to mutated genes detected in the HNSCC TCGA patient cohort indicating our 3D HNSCC cell cultures to be representative to a certain degree on the genetic level [38, 41, 42]. Interesting was also that responders and non-responders showed very similar mutation frequency of base exchanges, insertions, deletions, MNV and replacements, while protein-coding mutations revealed higher numbers in non-responders relative to responders. This observation strongly supported our hypothesis that, compared to responders, non-responders express a higher rate of functional mutations putatively eliciting resistance.

Taking the protein-coding gene mutations into account, the *in-vitro* identified mutational profiles of responder and non-responder cells allowed stratification of HNSCC patients. The 475 mutated genes unique for responders, 733 for non-responders and 318 overlapping genes provided a significant basis to categorize HNSCC patients and allocate prosurvival, promitotic signaling pathways such as ErbB, TGF-β and adherens junction signaling to non-responders. Conclusively, our whole exome data has the potential to predictively discriminate between HNSCC patients for different combinations of therapy. Our work pinpoints that a combination of genetic characterization before and during therapy has the greatest power to personalize cancer therapy. From the exome data, we also deduced novel potential targets whose deactivation might result in an enhanced cell kill per se or enhance HNSCC cell radiosensitivity.

Evaluating the data from the cytotoxicity and radiosensitization screen provided intriguing insights. As we have selected the most promising candidates by a combination of published key tumor criteria, the great overlap and independence from AIIB2 and Cetuximab of ineffective and effective targeting approaches between the different treatment groups were astonishing. This finding clearly argues for basic resistance mechanisms detectable prior to treatment. By reviewing the list of genes without impact like ERBB3, CASP8, RAF1 and KRAS (FaDu) as well as KEAP1, KRAS, RAF1 and FANCD2 (SAS), one finds tumor drivers ranked among the top 100 mutated genes in HNSCC [41, 42]. Further evaluation is required to provide answers to the connection of gene mutation frequency and functionality. This is, however, not only true for the ineffective candidates but also for the effective ones like ARID1B, RB1 and RHOA (FaDu) and EP300, ETV1 and CASC5 (SAS). Concerning radiosensitization, the list of effective genes consisted of KEAP1 and RAF1 (FaDu) and MTOR and KEAP1 (SAS). Consequently, we took mTOR and KEAP1 targeting further using pharmacological inhibitors. While we intentionally anticipated generating more supportive data from our broad-spectrum phosphoproteome array for novel target identification on top of whole exome sequencing, merely mTOR showed elevated activating phosphorylations in FaDu and SAS non-responders compared to UTSCC14, UTSCC15 and UTSCC45 responders.

Intriguing to us was to observe a significant reduction in HNSCC cell resistance to AIIB2/Cetuximab-mediated radiosensitization through Everolimus and ML344. HNSCC is the sixth most common malignancy worldwide. As a major public health concern, HNSCC arises at different places including oral cavity, larynx, and pharynx with an incidence of approximate 600,000 patients each year [38]. Highly critical is that only 40 to 50% survive more than 5 years; a rate, which is stable for over 3 decades [43]. Thus, novel therapies are desperately needed as already clinically approved strategies such as EGFR-targeting seem to be potent only in specific subpopulations when considering the equipotent chemotherapeutic cisplatin [44, 45]. In contrast to KEAP1, which we identified as novel target, mTOR has been suggested also by others based on the PI3K/mTOR pathway as the most frequently activated cascade for cancer initiation and progression [46, 47]. PI3Ks are not only the highest mutated genes but various genetic and epigenetic modifications coordinated with PI3K mutations elicit sustained activation of this pathway. Moreover, EGFR signaling found to be hyperactive in over 90% of HNSCC lesions is connected to PI3K/AKT and mTOR signaling [48]. mTOR is documented to regulate fundamental cell processes such as embryonic development, homeostasis, tumor growth and metabolism [49]. In line with our understanding on the central role of mTOR in tumor growth, we state that mTOR targeting – here with the clinically approved drug Everolimus – is an effective und unprecedented strategy to sensitize distinct resistant cancer cell populations to a β1 integrin- and EGFR-deactivating therapy.

Much less is known about KEAP1. Stacy and colleagues reported that KEAP1 is overexpressed in HNSCC relative to normal tissue. This overexpression could be responsible for enhanced expression of the transcription factor nuclear factor E2 p45-related factor 2 (Nrf2) [50]. This finding is interesting in the context of establishing the four molecular classes of HNSCC (basal, mesenchymal, atypical, classical). Here, a deregulated KEAP1 oxidative stress pathway, in addition to the preference of other oncogenes such as PIK3CA and EGFR, was observed [51]. Mechanistically, Nrf2 and KEAP1 negatively interact via a CUL3/RBX E3-ubiquitin ligase complex [52]. Overexpression and hypermethylation of KEAP1 elicit a disruption of this protein complex in 64% of HNSCC patients with poor outcome. In prostate cancer, Zhang and colleagues showed that KEAP1 has loss-of-function mutations providing a therapeutic potential for Nrf2 targeting [53]. Likewise in lung cancer, deletion of KEAP1 promotes cancer aggressiveness, metastasis and resistance to oxidative stress as produced by radiotherapy [54]. While future analyses are warranted to unravel the resistance mechanisms driven by KEAP1, our data support a key role of KEAP1 in the cellular radiation and DNA damage response of HNSCC cells.

In summary, our data pinpoint the added value of genetic biomarker identification after selection for cancer subgroup responsiveness to targeted therapies. To actually translate these observations into the clinic, we need to connect the preclinical with the clinical data on specific predictive biomarkers before, during and after therapy as well as long-term outcome. This is differentially challenging as (i) EGFR-targeting, but not β1 integrin targeting, is already administered in HNSCC patients and (ii) Cisplatin/radiotherapy compared with Cetuximab/radiotherapy are equipotent minimizing the patient number treated with Cetuximab/radiotherapy. Despite these desperately warranted translation studies for predictive biomarker identification, our study suggests that genetic characterization can be highly supportive to develop potent, yet unprecedented multi-targeting strategies to precisely inhibit persisting resistance mechanisms in certain cancer cell subpopulations prior to therapy.

## MATERIALS AND METHODS

### Antibodies

Antibodies were purchased as indicated: β1 integrin (BD Biosciences), EGFR, Erk1/2, p-Erk1/2 (T202/Y204), FAK, p-FAK (Y397), mTOR, p-mTOR (S2448) (Cell Signaling); KEAP1 (GeneTex), β-Actin (Sigma), p-EGFR (Y1068) (Thermo Fisher Scientific); HRP-conjugated donkey anti-rabbit and sheep anti-mouse secondary antibodies (GE Healthcare).

### Cell culture

Human head and neck squamous cell carcinoma (HNSCC) cell lines UTSCC14, UTSCC15, UTSCC45 and SAS were kindly provided by R. Grenman (Turku University Central Hospital, Finland). FaDu cells were purchased from ATCC. Origin and stability of the cells were routinely monitored by short tandem repeat analysis (microsatellites). Cells were cultured in Dulbecco's modified Eagle's medium (DMEM; Thermo Fisher Scientific) containing glutamax-I supplemented with 10% fetal calf serum (FCS; PAA) and 1% non-essential amino acids (Sigma) at 37° C in a humidified atmosphere containing 8.5% CO_2_. In all experiments, asynchronously growing cells were used.

### DNA isolation

For DNA isolation, cells were cultured in 0.5 mg/ml lrECM for 4 days. Cells were segregated using Trypsin/EDTA and DNA was isolated using the NucleoSpin^®^ Tissue kit (Macherey & Nagel) according to the manufacturer's protocol. DNA concentration was measured with Qubit^®^ 2.0 (Thermo Fisher Scientific) and 1 μg DNA was utilized for whole exome sequencing.

### Whole exome sequencing

Genomic DNA (1 μg) was sheared to 100 – 400 bp using a Covaris S2 (Covaris, Woburn, Massachusetts, USA). Sheared DNA was subjected to Illumina paired-end DNA library preparation. Pools of 8 differently indexed NGS libraries were enriched for target sequences using the SureSelect XT2 chemistry V5 Human All Exon + UTR (Agilent Technologies, Santa Clara, CA, USA) according to the manufacturer's recommendations. Enriched libraries were sequenced using the HiSeq 2000 platform (Illumina) as paired-end 75 base reads leading to on average approx. 90 mio reads per sample.

### Reads mapping and mutations detection

Whole exome sequencing (WES) raw data of cell lines were mapped against human genome reference sequence (hg19-Ensembl) using CLC Biomedical Genomics Workbench v.3.51 (Qiagen, Aarhus, Denmark) (CLC BMW) with following parameters: match score 1; mismatch cost 2; affine gap cost (Insertion/deletion open cost 6; insertion/deletion extend cost 1); length fraction 0.5; similarity fraction 0.8. Reads that mapped equally at multiple sites were discarded. Variants were detected via low frequency variant detection tool of CLC BMW using default parameters (minimum coverage: 10; minimum count: 2; minimum frequency: 1.0%; relative read direction filter). Only variants with a minimum frequency of at least 30%, a minimum coverage of at least 10 reads and a minimum CLC quality parameter QUAL of 200 were used for subsequent analysis. Variants were annotated with ClinVar, COSMIC v.78 and ExAC v0.3 variant information. Variant effect on genes was determined via CLC BMW using human Ensembl gene information (hg19). Classification of variants into missense variants and truncation variants (including nonsense variants, frameshift variants and variants potentially causing splice site truncation) were conducted. Potential splice site variants were detected in ± 2 nucleotides from corresponding exon. Variants were manually evaluated and pathogenic variants were identified. Cancer related genes were identified on the basis of the COSMIC CENSUS list (http://cancer.sanger.ac.uk/census/). Copy number variations (CNVs) were investigated via CLC BMW using the CNV-detection tool with default parameters in cell line WES mappings using the remaining cell line mappings as control datasets. Somatic signatures were determined using the R package SomaticSignatures [55]. For investigated cell lines, all genome-wide occurring SNVs were used for calculating individual mutations spectrum.

### Signaling pathway analysis

Exclusively mutated genes identified in at least one responder or in at least one non-responder cell line were mapped to known cancer signaling pathways using annotations form [56]. The number of mutated genes per pathway was counted separately for responder and non-responder and the significance of gene enrichment was determined using Fisher's exact test.

### TCGA HNSCC data analysis

Curated somatic mutations of 279 patients were downloaded from the TCGA data portal (download date: 18-Feb-2016). Mutation frequencies of genes found to be exclusively mutated in at least one responder or in at least one non-responder cell line were determined for the TCGA patients. Similarity of individual TCGA patients to responder and non-responder profiles were computed based on the distance of each patient-specific gene mutation profile to the responder and non-responder profile. In more detail, focusing on exclusively mutated genes of responders and non-responders that were mutated in at least 8% of patients, we created a potential responder mutation profile that contained a value of one for each gene that was exclusively mutated in at least one responder cell line and a value of zero for each gene that was exclusively mutated in at least one non-responder cell line. In analogy, we created a potential non-responder mutation profile. Next, we focused on the genes that were exclusively found to be mutated in responders or non-responders and computed for each patient a corresponding patient-specific mutation profile by coding each patient-specific gene mutation by a value of one and no mutation by a value of zero. We next determined the Manhattan distance of each patient to the responder and to the non-responder profile and computed the ratio of responder to non-responder distance. A ratio less than one indicated that a patient is more similar to the responder profile, whereas a ratio greater one indicated that a patient is more similar to the non-responder profile. We used this ratio to sort patients according to their similarity from responder to non-responder and plotted the corresponding mutation profiles using the standard R heatmap function.

### 3D colony formation assay

Twenty-four genes were depleted using specific endoribonuclease-prepared siRNAs (esiRNAs, Eupheria) (Supplementary Table 2) [57]. Seventy thousand cells per 24 well were seeded and incubated for 24 h at 37° C. For transfection, 20 nM esiRNA was mixed with Oligofectamine and OptiMEM (both Thermo Fisher Scientific). After 20 min of incubation at room temperature, transfection reagent was added to the cells and incubated for 8 h at 37° C. The reaction was stopped by adding OptiMEM plus 20% FCS. After 16 h, cells were seeded for 3D colony formation assay as published [58]. In brief, cells were mixed with 0.5 mg/ml laminin-rich extracellular matrix (lrECM (Matrigel™; BD Biosciences) and plated into 96 well plates. Twenty-four hours later, cells were treated with AIIB2 and/or Cetuximab (or IgG isotype control) and irradiated with 6 Gy X-rays 1 h later. After an incubation period of 8 days, cell clusters with a minimum of 50 cells were counted microscopically. Top hits in IgG, AIIB2, Cetuximab and AIIB2/Cetuximab treated FaDu and SAS cells with a surviving fraction < 0.5 and a *p*-value < 0.05 were analyzed for putative protein-protein interactions and a molecular interaction network was created using Cytoscape with STRING-app (Cytoscape Consortium [59]).

### Radiation exposure

X-ray irradiation was delivered at room temperature using single doses of 200 kV X-rays filtered with 0.5 mm Cu (Yxlon Y.TU 320; Yxlon, Hamburg, Germany). The dose-rate was approximately 1.3 Gy/min at 20 mA. The absorbed dose was measured using a Duplex dosimeter (PTW, Freiburg, Germany). Applied single doses ranged from 2 to 6 Gy X-rays.

### Inhibitory antibodies and inhibitor treatment

For β1 integrin blocking, 10 μg/ml of the inhibitory monoclonal antibody AIIB2 was used as published [12]. Epidermal growth factor receptor inhibition was accomplished using Erbitux^®^ (Cetuximab; 5 μg/ml; Merck). 3D lrECM cell cultures were treated with AIIB2, Cetuximab or IgG isotype control for 1 h prior to irradiation. Where indicated, cells were additionally treated with Rad001 (Everolimus; 0.01–14 μM, Tocris) or ML334 (3–50 μM, Axon Medchem) 1 h prior to irradiation. DMSO was used as control. After 24 h, cells were washed with fresh culture medium.

### Phosphoproteome analysis

The Phospho Explorer Antibody Microarray was conducted by Full Moon BioSystems Inc. as published [12, 60]. Briefly, whole-cell lysates were prepared from UTSCC14, UTSCC15, UTSCC45, SAS and FaDu cell cultures grown for 4 days in 0.5 mg/ml lrECM. Cells were harvested using modified RIPA buffer (50 mM Tris-HCl (pH7.4), 1% Nonidet-P40, 0.25% sodium deoxycholate, 150 mM NaCl, 1 mM EDTA, Complete protease inhibitor cocktail, 1 mM NaVO_4_, 2 mM NaF). Lysates were transferred to Full Moon BioSystems Inc. on dry ice. The array consists of antibodies against 342 proteins and 606 phospho-sites. Proteins were labeled with biotin and placed on preblocked microarray slides. After washing, detection of total and phosphorylated proteins was conducted using Cy3-conjugated streptavidin. Expression of phosphorylated proteins was normalized to corresponding total protein expression. A table with results of the whole analysis is given in the supplement (Supplementary Table 5).

### Total protein extracts and western blotting

Whole cell lysates were harvested from cells treated with AIIB2, Cetuximab, AIIB2/Cetuximab or IgG. Cells lysis and SDS-PAGE/Western Blotting were performed as previously published [61]. After SDS-PAGE and transfer of proteins onto nitrocellulose membranes (GE Healthcare), probing of specific proteins was accomplished using indicated primary antibodies and horseradish peroxidase-conjugated donkey anti-rabbit and sheep anti-mouse antibodies (GE Healthcare). Enhanced chemiluminescent reagent (GE Healthcare) was used for detection of proteins on X-ray films (GE Healthcare) and ImageJ for densitometry.

### Data analysis

Means ± SEM of at least three independent experiments were calculated with reference to controls defined in total numbers or 1.0. For statistical significance analysis of clonogenic survival and densitometry two-sided Student's *t*-test was performed using Excel (Microsoft). *P*-value of less than 0.05 was considered statistically significant.

## SUPPLEMENTARY MATERIALS FIGURES AND TABLES










