# Supplementary material for: Whole exome sequencing identifies mTOR and KEAP1 as potential targets for radiosensitization of HNSCC cells refractory to EGFR and β1 integrin inhibition
Source: Oncotarget. 2018 Apr 6;9(26):18099–114. doi: 10.18632/oncotarget.24266 (PMC5915060; doi:10.18632/oncotarget.24266)
Supplement: Supplementary file 1 [file oncotarget-09-18099-s001.pdf]

Whole exome sequencing identifies mTOR and KEAP1 as potential targets for radiosensitization of HNSCC cells refractory to EGFR and β1 integrin inhibition

SUPPLEMENTARY MATERIALS

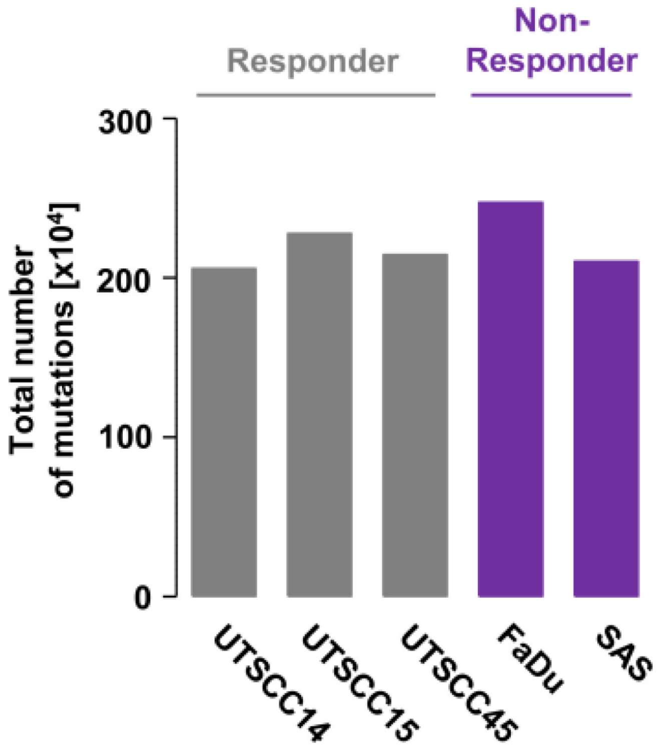

Supplementary Figure 1: Mutations found in responder and non-responder cells. Total number of non-coding and coding mutations detected in 3D grown responder (grey) and non-responder (purple) HNSCC cell lines.

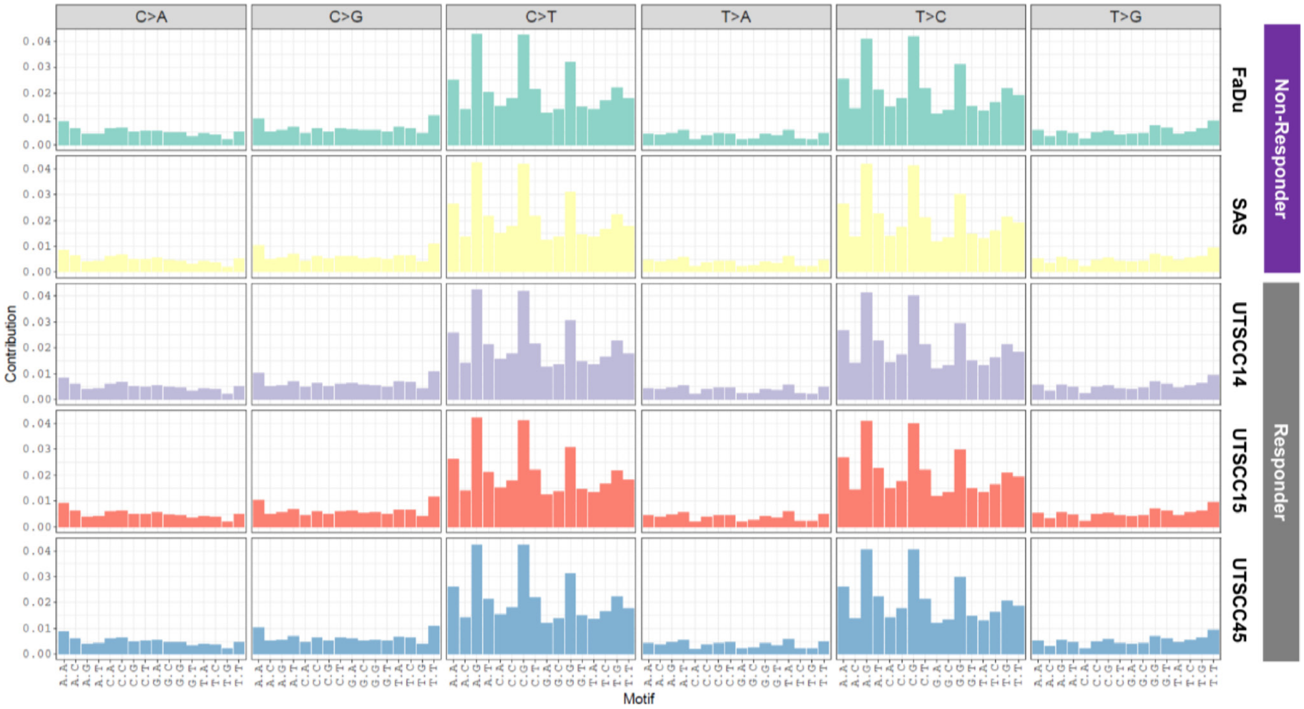

Supplementary Figure 2: Mutational motifs of responder and non-responder cells. Graphical representation of signatures identified in 3D lrECM-based UTSCC14, UTSCC15 and UTSCC45 (responders) as well as FaDu and SAS (non-responders) cell cultures. Mutations were identified by whole-exome sequencing.

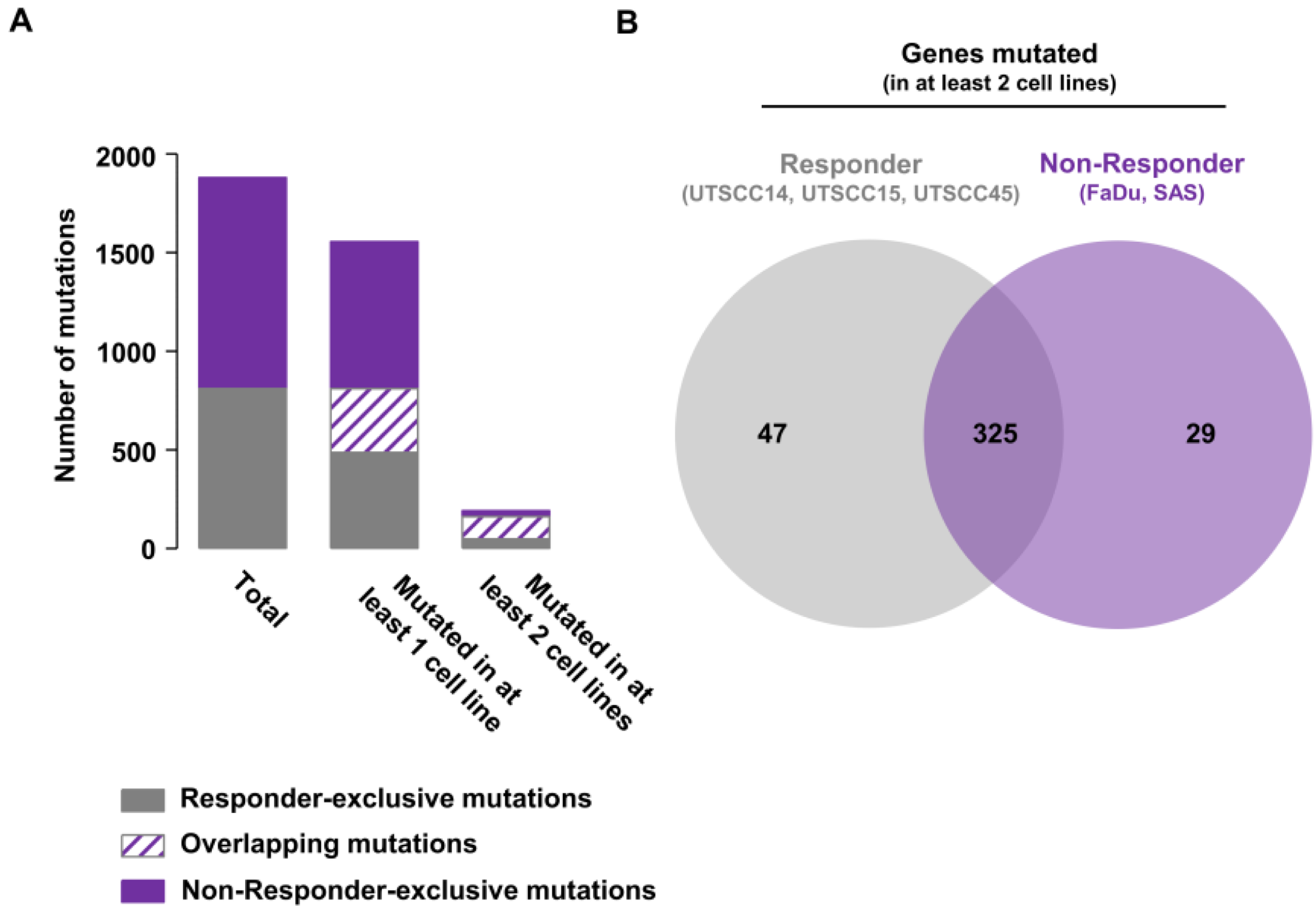

Supplementary Figure 3: Mutational profiles of responder and non-responder cells. (**A**) Number of coding mutations detected in 3D grown responder (grey) and non-responder (purple) HNSCC cell lines. (**B**) Venn diagram analysis of all mutated genes showing shared mutations and exclusively mutated genes in the responder or non-responder groups.

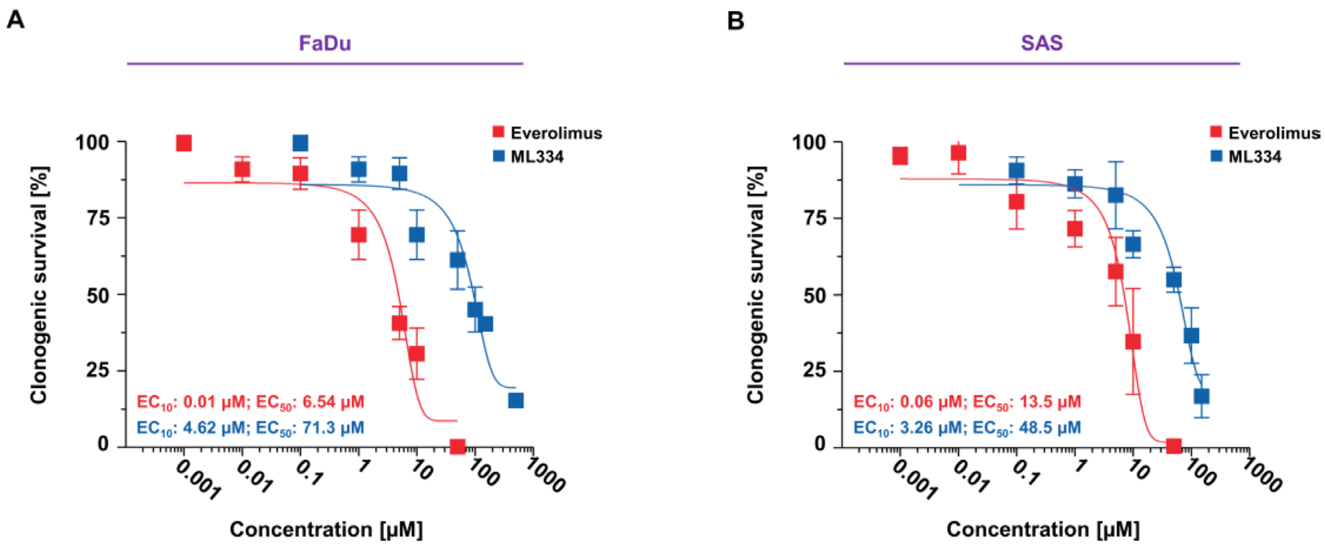

Supplementary Figure 4: Cytotoxicity of Everolimus and ML334. (**A and B**) Everolimus (mTORi) and ML334 (KEAP1i) cytotoxicity and corresponding EC10 and EC50 values in FaDu (A) and SAS (B) cells (colony formation assay). Results show mean ± SEM (*n* = 3).

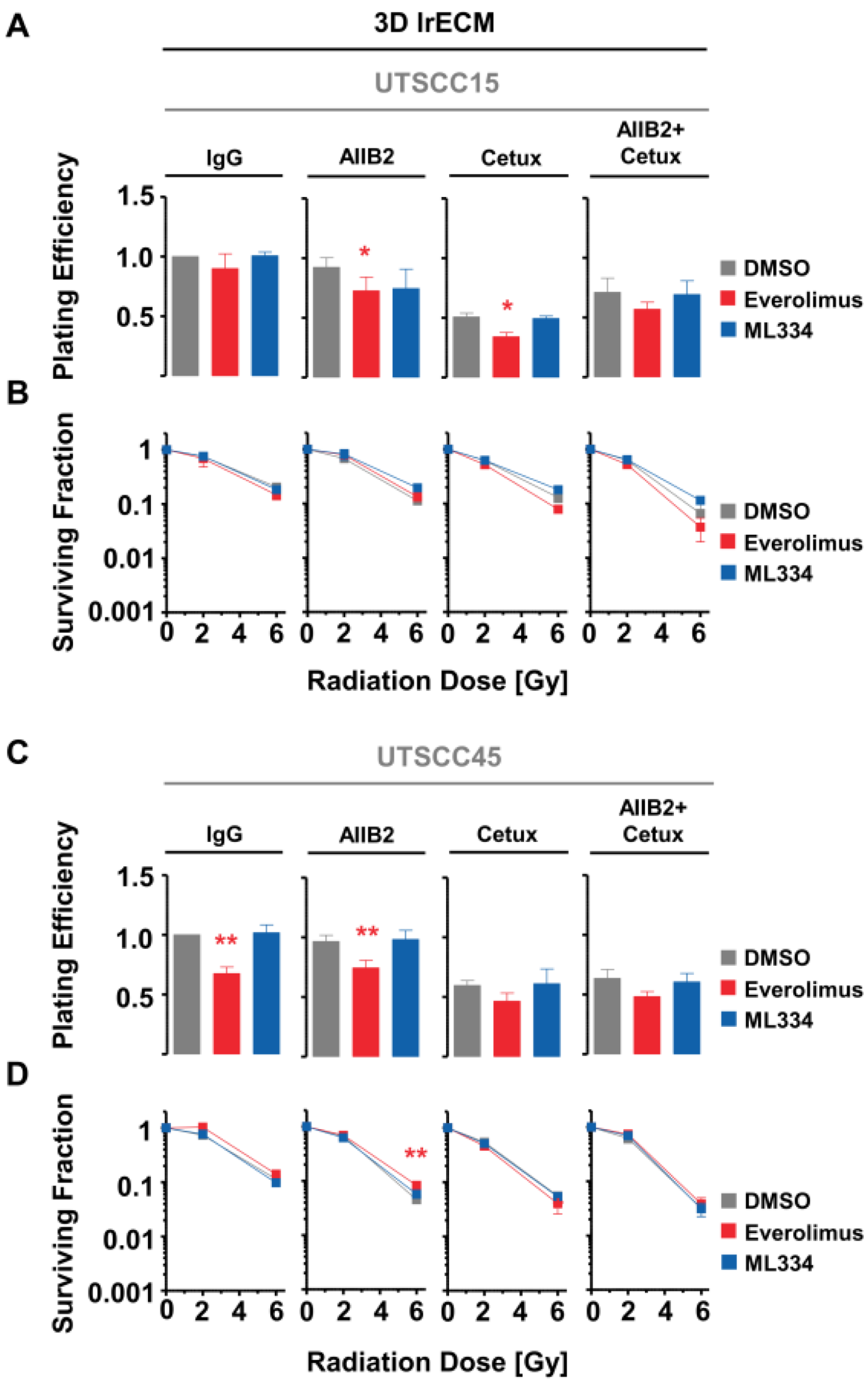

Supplementary Figure 5: Pharmacological inhibition of KEAP1 and mTOR fails to further radiosensitize AIIB2/ Cetuximab treated UTSCC15 and UTSCC45 responder cells. (**A**) Cytotoxicity measured as clonogenic survival and (**B**) clonogenic radiation survival of UTSCC15 cells upon treatment with 10 nM Everolimus (mTOR inhibitor) or 3.3 µM ML334 (KEAP1i) simultaneously to AIIB2, Cetuximab or AIIB2/Cetuximab plus 2–6 Gy X-rays (DMSO, IgG and 0 Gy as control). (**C**) Cytotoxicity measured as clonogenic survival and (**D**) clonogenic radiation survival of UTSCC45 cells upon treatment with 10 nM Everolimus (mTOR inhibitor) or 3.3 µM ML334 (KEAP1i) simultaneously to AIIB2, Cetuximab or AIIB2/Cetuximab plus 2–6 Gy X-rays (DMSO, IgG and 0 Gy as control).

Supplementary Table 1: Number and type of mutations detected in HNSCC responder (UTSCC14, UTSCC15, UTSCC45) and non-responder (FaDu, SAS) cells using whole exome sequencing

Type	UT14	UT15	UT45	FaDu	SAS
**A>C**	5750	6429	5951	7007	5950
**A>G**	21434	24778	22636	27695	21975
**A>T**	4227	4744	4416	5108	4388
**C>A**	5307	5882	5487	6696	5423
**C>G**	6627	7395	7027	8445	6664
**C>T**	21336	24569	22746	27563	22017
**G>A**	21383	24398	22730	27672	21921
**G>C**	6580	7385	6843	8376	6572
**G>T**	5224	5984	5451	6777	5396
**T>A**	4367	4841	4518	5184	4353
**T>C**	21686	24972	22580	27760	22247
**T>G**	5620	6448	5900	7037	5676
**Insertion**	11147	13135	11830	14858	11758
**Deletion**	13702	16251	14728	18766	14460
**SNV**	129541	147825	136285	165320	132582
**MNV**	3153	3514	3459	3685	3360
**Replacement**	527	592	551	680	574
**Total**	158070	181317	166853	203309	162734

Supplementary Table 2: ID, gene name and sequence of used esiRNAs. See Supplementary_Table_2

Supplementary Table 3: Results of the esiRNA screen in 3D grown FaDu cells for the indicated treatments. *P*-values are referring to the corresponding RLUC controls. See Supplementary_Table_3

Supplementary Table 4: Results of the esiRNA screen in 3D grown SAS cells for the indicated treatments. *P*-values are referring to the corresponding RLUC controls. See Supplementary_Table_4

Supplementary Table 5: Data of the ratio of phosphorylated protein to non-phosphorylated protein levels from phosphoproteome array of 3D cultured UTSCC14, UTSCC15, UTSCC45, SAS and FaDu cells. See Supplementary_ Table_5
