# Supplementary material for: Whole exome sequencing identifies mTOR and KEAP1 as potential targets for radiosensitization of HNSCC cells refractory to EGFR and β1 integrin inhibition
Source: Oncotarget. 2018 Apr 6;9(26):18099–114. doi: 10.18632/oncotarget.24266 (PMC5915060; doi:10.18632/oncotarget.24266)
Supplement: Supplementary file 2 [file oncotarget-09-18099-s002.doc]

**Supplementary Table 2**

**Table S2.** ID, gene name and sequence of used esiRNAs.

| **ID** | **Gene name** | **Sequence** |
| --- | --- | --- |
| RLUC | RLUC | GATAACTGGTCCGCAGTGGTGGGCCAGATGTAAACAAATGAATGTTCTTGATTCATTTATTAATTATTATGATTCAGAAAAACATGCAGAAAATGCTGTTATTTTTTTACATGGTAACGCGGCCTCTTCTTATTTATGGCGACATGTTGTGCCACATATTGAGCCAGTAGCGCGGTGTATTATACCAGACCTTATTGGTATGGGCAAATCAGGCAAATCTGGTAATGGTTCTTATAGGTTACTTGATCATTACAAATATCTTACTGCATGGTTTGAACTTCTTAATTTACCAAAGAAGATCATTTTTGTCGGCCATGATTGGGGTGCTTGTTTGGCATTTCATTATAGCTATGAGCATCAAGATAAGATCAAAGCAATAGTTCACGCTGAAAGTGTAGTAGATGTGATTGAATCATGGGATGAATGG |
| HU-11443-1 | KRAS | AGGCCTGCTGAAAATGACTGAATATAAACTTGTGGTAGTTGGAGCTGGTGGCGTAGGCAAGAGTGCCTTGACGATACAGCTAATTCAGAATCATTTTGTGGACGAATATGATCCAACAATAGAGGATTCCTACAGGAAGCAAGTAGTAATTGATGGAGAAACCTGTCTCTTGGATATTCTCGACACAGCAGGTCAAGAGGAGTACAGTGCAATGAGGGACCAGTACATGAGGACTGGGGAGGGCTTTCTTTGTGTATTTGCCATAAATAATACTAAATCATTTGAAGATATTCACCATTATAGAGAACAAATTAAAAGAGTTAAGGACTCTGAAGATGTACCTATGGTCCTAGTAGGAAATAAATGTGATTTGCCTTCTAGAACAGTAGACACAAAACAGGCTCAGGA |
| HU-15209-1 | CASP8 | TCCAAATGCAAACTGGATGATGACATGAACCTGCTGGATATTTTCATAGAGATGGAGAAGAGGGTCATCCTGGGAGAAGGAAAGTTGGACATCCTGAAAAGAGTCTGTGCCCAAATCAACAAGAGCCTGCTGAAGATAATCAACGACTATGAAGAATTCAGCAAAGAGAGAAGCAGCAGCCTTGAAGGAAGTCCTGATGAATTTTCAAATGACTTTGGACAAAGTTTACCAAATGAAAAGCAAACCTCGGGGATACTGTCTGATCATCAACAATCACAATTTTGCAAAAGCACGGGAGAAAGTGCCCAAACTTCACAGCATTAGGGACAGGAATGGAACACACTTGGATGCAGGGGCTTTGACCACGACCTTTGAAGAGCTTCATTTTGAGATCAAGCCCC |
| HU-10164-1 | FRAP1 | CCATTCATTTTGGGGAACAGAAGATCCATAACTTTAGAAATACGGGTTTTGACTTAACTCACAAGAGAACTCATCATAAGTACTTGCTGATGGAAGAATGACCTAGTTGCTCCTCTCAACATGGGTACAGCAAACTCAGCACAGCCAAGAAGCCTCAGGTCGTGGAGAACATGGATTAGGATCCTAGACTGTAAAGACACAGAAGATGCTGACCTCACCCCTGCCACCTATCCCAAGACCTCACTGGTCTGTGGACAGCAGCAGAAATGTTTGCAA |
| HU-05013-1 | RAF1 | CCAGAGTGCTGTGCAGTGTTCAGACTTCTCCACGAACACAAAGGTAAAAAAGCACGCTTAGATTGGAATACTGATGCTGCGTCTTTGATTGGAGAAGAACTTCAAGTAGATTTCCTGGATCATGTTCCCCTCACAACACACAACTTTGCTCGGAAGACGTTCCTGAAGCTTGCCTTCTGTGACATCTGTCAGAAATTCCTGCTCAATGGATTTCGATGTCAGACTTGTGGCTACAAATTTCATGAGCACTGTAGCACCAAAGTACCTACTATGTGTGTGGACTGGAGTAACATCAGACAACTCTTATTGTTTCCAAATTCCACTATTGGTGATAGTGGAGTCCCAGCACTACCTTCTTTGACTATGCGTCGTATGCGAGAGTCTGTTTCCAGGATGCCTGTT |
| HU-03096-1 | ERBB3 | TGGAACTGTGCACAAAGGAGTGTGGATCCCTGAGGGTGAATCAATCAAGATTCCAGTCTGCATTAAAGTCATTGAGGACAAGAGTGGACGGCAGAGTTTTCAAGCTGTGACAGATCATATGCTGGCCATTGGCAGCCTGGACCATGCCCACATTGTAAGGCTGCTGGGACTATGCCCAGGGTCATCTCTGCAGCTTGTCACTCAATATTTGCCTCTGGGTTCTCTGCTGGATCATGTGAGACAACACCGGGGGGCACTGGGGCCACAGCTGCTGCTCAACTGGGGAGTACAAATTGCCAAGGGAATGTACTACCTTGAGGAACATGGTATGGTGCATAGAAACCTGGCTGCCCGAAACGTGCTACTCAAGTCACCCAGTCAGGTTCAGGTGGCAGATTTTGGTGTGGCTGACCT |
| HU-00715-1 | P2RY8 | TGACCTGTATCAGCGTGGAGCGCTTCCTGGGGGTCCTGTACCCGCTCAGCTCCAAGCGCTGGCGCCGCCGTCGTTACGCGGTGGCCGCGTGTGCAGGGACCTGGCTGCTGCTCCTGACCGCCCTGTCCCCGCTGGCGCGCACCGATCTCACCTACCCGGTGCACGCCCTGGGCATCATCACCTGCTTCGACGTCCTCAAGTGGACGATGCTCCCCAGCGTGGCCATGTGGGCCGTGTTCCTCTTCACCATCTTCATCCTGCTGTTCCTCATCCCGTTCGTGATCACCGTGGCTTGTTACACGGCCACCATCCTCAAGCTGTTGCGCACGGAGGAGGCGCACGGCCGGGAGCAGCGGAGGCGCGCGGTGGGCCTGGCCGCGGTGGTCTTGCTGGCCTTTGTCACCTGCTTCGCCCCCAACAACTTCGTGCTCCTGGCGCACATCGTGAGCCGCCTGTTCTACGGCAAGAGCTACTACCACGTGTACAAGCTCACGCTGTGTCTCAGCTGCCTCAACAACTGTCTGGACCCGTTTGTTT |
| HU-12790-1 | ARID1B | AAGCGATGCATCTGTGTGTCCAATATTGTCCGTAGCTTGTCATTCGTGCCTGGCAATGATGCCGAAATGTCCAAACATCCAGGCCTGGTGCTGATCCTGGGGAAGCTGATTCTTCTTCACCACGAGCATCCAGAGAGAAAGCGAGCACCGCAGACCTATGAGAAAGAGGAGGATGAGGACAAGGGGGTGGCCTGCAGCAAAGATGAGTGGTGGTGGGACTGCCTCGAGGTCTTGAGGGATAACACGTTGGTCACGTTGGCCAACATTTCCGGGCAGCTAGACTTGTCTGCTTACACGGAAAGCATCTGCTTGCCAATTTTGGATGGCTTGCTGCACTGGATGGTGTGCCCGTCTGCAGAGGCACAAGATCCCTTTCCAACTGTGGGACCCAACTCGGTCCTGTCGCCTCAGAGACTTGTGCTGGAGACCCTCTGTAAACTCAGTATCCAGGACAATAATGTGGACCTGATCTTGGC |
| HU-12271-1 | ARHGEF12 | CAGTGCAGCTGTTTCCAGAGCATTGAATTACTAAAATCTCGCCCGGCTCATTTGGCTGTTTTCTTACACCATGTAGTTTCACAATTTGACCCTGCGACTTTGCTCTGTTATCTCTATTCAGACCTGTATAAACATACCAATTCCAAAGAAACTCGTCGCATCTTCCTTGAGTTTCATCAGTTCTTTCTAGATCGATCAGCACACCTGAAAGTTTCTGTTCCTGATGAAATGTCTGCAGATCTAGAAAAGAGAAGACCTGAGCTCATTCCTGAGGATCTGCATCGCCACTATATCCAAACTATGCAAGAAAGAGTCCATCCAGAAGTTCAAAGGCACTTAGAAGATTTTCGGCAGAAACGTAGTATGGGACTGACCTTGGCTGAAAGCGAGCTGACTAAACTTGATGCAGAGCGAGACA |
| HU-07157-1 | TRAF7 | GTGGCTCCTCTGACAAGACCATCAAGGTGTGGGACACATGTACCACCTACAAGTGTCAGAAGACACTGGAGGGCCATGATGGCATCGTGCTGGCTCTCTGCATCCAGGGGTGCAAACTCTACAGCGGCTCTGCAGACTGCACCATCATTGTGTGGGACATCCAGAACCTGCAGAAGGTGAACACCATCCGGGCCCATGACAACCCGGTGTGCACGCTGGTCTCCTCACACAACGTGCTCTTCAGCGGCTCCCTGAAGGCCATCAAGGTCTGGGACATCGTGGGCACTGAGCTGAAGTTGAAGAAGGAGCTCACAGGCCTCAACCACTGGGTGCGGGCCCTGGTGGCTGCCCAGAGCTACCTGTACAGCGGCTCCTACCAGACAATCAAGATCTGGGACATCCGAACCCTTGACTGCATCCACGTCCTGCAGACGTCTGGTGGCAGCGTCTACT |
| HU-00153-1 | GPC3 | GCTCTTACTGCCAGGGACTGATGATGGTTAAACCCTGTGGCGGTTACTGCAATGTGGTCATGCAAGGCTGTATGGCAGGTGTGGTGGAGATTGACAAGTACTGGAGAGAATACATTCTGTCCCTTGAAGAACTTGTGAATGGCATGTACAGAATCTATGACATGGAGAACGTACTGCTTGGTCTCTTTTCAACAATCCATGATTCTATCCAGTATGTCCAGAAGAATGCAGGAAAGCTGACCACCACTATTGGCAAGTTATGTGCCCATTCTCAACAACGCCAATATAGATCTGCTTATTATCCTGAAGATCTCTTTATTGACAAGAAAGTATTAAAAGTTGCTCATGTAGAACATGAAGAAACCTTATCCAGCCGAAGAAGGGAACTAATTCAGAAGTTGAAGTCTTTCATCAGCTTCTATAGTGCTTTGCCTGGCTACAT |
| HU-02926-1 | FANCD2 | TCCGACTTGACCCAAACTTCCTATTGAAGGTTCGCCAGTTGGTGATGGATAAGTTGTCGTCTATTAGATTGGAGGATTTACCTGTGATAATAAAGTTCATTCTTCATTCCGTAACAGCCATGGATACACTTGAGGTAATTTCTGAGCTTCGGGAGAAGTTGGATCTGCAGCATTGTGTTTTGCCATCACGGTTACAGGCTTCCCAAGTAAAGTTGAAAAGTAAAGGACGAGCAAGTTCCTCAGGAAATCAAGAAAGCAGCGGTCAGAGCTGTATTATTCTCCTCTTTGATGTAATAAAGTCAGCTATTAGATATGAGAAAACCATTTCAGAAGCCTGGATTAAGGCAATTGAAAACACTGCCTCAGTATCTGAACACAAGGTGTTTGACCTGGTGATGCTTTTCATCATCTATAGCACCAATACTCAGACAAAGAAGTACATTGACAGGGTGCTAAGAAATAAGATTCGATCAGGCTGCATT |
| HU-01356-1 | MSH2 | TTCATGGCTGAAATGTTGGAAACTGCTTCTATCCTCAGGTCTGCAACCAAAGATTCATTAATAATCATAGATGAATTGGGAAGAGGAACTTCTACCTACGATGGATTTGGGTTAGCATGGGCTATATCAGAATACATTGCAACAAAGATTGGTGCTTTTTGCATGTTTGCAACCCATTTTCATGAACTTACTGCCTTGGCCAATCAGATACCAACTGTTAATAATCTACATGTCACAGCACTCACCACTGAAGAGACCTTAACTATGCTTTATCAGGTGAAGAAAGGTGTCTGTGATCAAAGTTTTGGGATTCATGTTGCAGAGCTTGCTAATTTCCCTAAGCATGTAATAGAGTGTGCTAAACAGAAAGCCCTGGAACTTGAGGAGTTTCAGTATATTGGAGAATCGCAAGGATATGATATCATGGAACCAGCAGCAAAGAAGTGCTATCTGGAAAGAGAGCAAGGTGAAAAAATTATTCAGGAGTTCCTGTCCAAGGTGAAACAAATGCCCT |
| HU-02353-1 | RB1 | TGCATGGCTCTCAGATTCACCTTTATTTGATCTTATTAAACAATCAAAGGACCGAGAAGGACCAACTGATCACCTTGAATCTGCTTGTCCTCTTAATCTTCCTCTCCAGAATAATCACACTGCAGCAGATATGTATCTTTCTCCTGTAAGATCTCCAAAGAAAAAAGGTTCAACTACGCGTGTAAATTCTACTGCAAATGCAGAGACACAAGCAACCTCAGCCTTCCAGACCCAGAAGCCATTGAAATCTACCTCTCTTTCACTGTTTTATAAAAAAGTGTATCGGCTAGCCTATCTCCGGCTAAATACACTTTGTGAACGCCTTCTGTCTGAGCACCCAGAATTAGAACATATCATCTGGACCCTTTTCCAGCACACCCTGCAGAATGAGTATGAACTCATGAGAGACAGGCATTTGGACC |
| HU-07049-1 | ETV1 | CCCCAGGCAGTTTTATGATGACACCTGTGTTGTCCCAGAAAAATTCGATGGAGACATCAAACAAGAGCCAGGAATGTATCGGGAAGGACCCACATACCAACGGCGAGGATCACTTCAGCTCTGGCAGTTTTTGGTAGCTCTTCTGGATGACCCTTCAAATTCTCATTTTATTGCCTGGACTGGTCGAGGCATGGAATTTAAACTGATTGAGCCTGAAGAGGTGGCCCGACGTTGGGGCATTCAGAAAAACAGGCCAGCTATGAACTATGATAAACTTAGCCGTTCACTCCGCTATTACTATGAGAAAGGAATTATGCAAAAGGTGGCTGGAGAGAGATATGTCTACAAGTTTGTGTGTGATCCAGAAGCCCTTTTCTCCATGGCCTTTCCAGATAATCAGCGTCCACTGCTGAAGACAGACATGGAACGTCACATCAACGAGGAGGACACAGTG |
| HU-13250-1 | PAX3 | AGCTCGGCGGTGTTTTTATCAACGGCAGGCCGCTGCCCAACCACATCCGCCACAAGATCGTGGAGATGGCCCACCACGGCATCCGGCCCTGCGTCATCTCGCGCCAGCTGCGCGTGTCCCACGGCTGCGTCTCCAAGATCCTGTGCAGGTACCAGGAGACTGGCTCCATACGTCCTGGTGCCATCGGCGGCAGCAAGCCCAAGCAGGTGACAACGCCTGACGTGGAGAAGAAAATTGAGGAATACAAAAGAGAGAACCCGGGCATGTTCAGCTGGGAAATCCGAGACAAATTACTCAAGGACGCGGTCTGTGATCGAAACACCGTGCCGTCAGTGAGTTCCATCAGCCGCATCCTGAGAAGTAAATTCGGGAAAGGTGAAGAGGAGGAGGCCGACTTGGAGAGGAAGGAGGCAGAGGAAAG |
| HU-07737-1 | RHOA | CGGTCTGGTCTTCAGCTACCCGCCTTCGTCTCCGAGTTTGCGACTCGCGGACCGGCGTCCCCGGCGCGAAGAGGCTGGACTCGGATTCGTTGCCTGAGCAATGGCTGCCATCCGGAAGAAACTGGTGATTGTTGGTGATGGAGCCTGTGGAAAGACATGCTTGCTCATAGTCTTCAGCAAGGACCAGTTCCCAGAGGTGTATGTGCCCACAGTGTTTGAGAACTATGTGGCAGATATCGAGGTGGATGGAAAGCAGGTAGAGTTGGCTTTGTGGGACACAGCTGGGCAGGAAGATTATGATCGCCTGAGGCCCCTCTCCTACCCAGATACCGATGTTATACTGATGTGTTTTTCCATCGACAGCCCTGATAGTTTAGAAAACATCCCAGAAAAGTGGACCCCAGAA |
| HU-15515-1 | EP300 | ATACCGAACCAAAGCCCTCTTTGCCTTTGAAGAAATTGATGGTGTTGACCTGTGCTTCTTTGGCATGCATGTTCAAGAGTATGGCTCTGACTGCCCTCCACCCAACCAGAGGAGAGTATACATATCTTACCTCGATAGTGTTCATTTCTTCCGTCCTAAATGCTTGAGGACTGCAGTCTATCATGAAATCCTAATTGGATATTTAGAATATGTCAAGAAATTAGGTTACACAACAGGGCATATTTGGGCATGTCCACCAAGTGAGGGAGATGATTATATCTTCCATTGCCATCCTCCTGACCAGAAGATACCCAAGCCCAAGCGACTGCAGGAATGGTACAAAAAAATGCTTGACAAGGCTGTATCAGAGCGTATTGTCCATGACTACAAGGATATTTTTAAACAAGCTACTGAAGATAGATTAACAAGTGCAAAGGAATTGCCTTATTTCGAGGGTGATTTCTGGCCCAATGTTCTG |
| HU-09715-1 | CASC5 | ATGCAACTGCTGTTTGTGGATCCAGTGATAATTATTCCTGTTTACCAAATGTTATTTCCTGTACTGATAATTTGGAGGGTAGTGCCATGCTCTTATGTGATAAAGATGAGGAAAAAGCCAATTATTGCCCAGTGCAAAATGATCTTGCTTATGCAAATGATTTTGCCAGTGAATATTACTTGGAATCTGAGGGACAGCCTCTCTCTGCTCCTTGTCCTTTGTTAGAGAAGGAAGAAGTTATTCAAACCAGTACCAAAGGACAGTTAGACTGTGTTATAACACTGCACAAAGATCAAGATCTGATTAAGGATCCACGAAATCTATTGG |
| HU-06543-1 | KEAP1 | ATCGATGGCCACATCTATGCCGTCGGCGGCTCCCACGGCTGCATCCACCACAACAGTGTGGAGAGGTATGAGCCAGAGCGGGATGAGTGGCACTTGGTGGCCCCAATGCTGACACGAAGGATCGGGGTGGGCGTGGCTGTCCTCAATCGTCTCCTTTATGCCGTGGGGGGCTTTGACGGGACAAACCGCCTTAATTCAGCTGAGTGTTACTACCCAGAGAGGAACGAGTGGCGAATGATCACAGCAATGAACACCATCCGAAGCGGGGCAGGCGTCTGCGTCCTGCACAACTGTATCTATGCTGCTGGGGGCTATGATGGTCAGGACCAGCTGAACAGCGTGGAGCGCTACGATGTGGAAACAGAGACGTGGACTTTCGTAGCCCCCATGAAGCACCGGCGAAGTGCCCTGGGGATCACTGTCCACCAGGGGAGAATCTACGTCCTTGGAGGCTATGATGGTCACA |
| HU-09903-1 | LAMA1 | GAACAGTGTGTGGTGGATGCAGCTCTGGAGTACGTTCCCGGCGCTCACCAGTTTGGTCTCACACAAAACAGCCATTTCATCTTGCCTTTTAATCAGTCGGCTGTCAGAAAGAAGCTCTCGGTTGAGCTAAGCATCCGCACGTTCGCCTCCAGCGGCCTGATTTACTACATGGCTCATCAGAACCAAGCAGACTACGCTGTGCTCCAGCTGCACGGGGGCCGCCTCCACTTCATGTTTGACCTTGGCAAAGG |
| HU-04158-1 | POLQ | TATATGGGCAGCACCTCTCCATCAAGGCATTTCTCTTCATTGGATATTGCAGTCTGCACAATTGAGAGAGCCAATGGTCTGATCAATCGCCTCATAGAGGAAAATAAGATGGATCTGTTAGGAATGGTGGTTGTGGATGAATTACATATGCTGGGAGACTCTCACCGAGGGTATCTGCTGGAACTTTTGCTGACCAAGATTTGCTATATTACTCGGAAATCAGCATCTTGTCAGGCAGATCTAGCCAGTTCTCTGTCTAATGCTGTGCAAATCGTTGGCATGAGTGCTACCCTTCCTAATTTGGAGCTTGTGGCTTCCTGGTTGAATGCTGAACTCTACCATACCGACTTTCGCCCTGTACCGCTTTTGGAGTCAGTAAAAGTTGGAAATTCCATATATGACTCTTCAATGAAACTTGTGAGGGAATTTGAGCCCATGCTACAAGTGAAGGGAGATGAGGACCA |
| HU-09805-1 | GPR98 | CCGTGTGACATTGGTTTCTGCAATTCCTGGAGATGGGAAGCTAGGCTCAACTCCTACCAGTGGTGCAAGCATAGATCCTGAAAAGGAAACGACTGATATCACCATCAAAGCTAGTGATCATCCATATGGCTTGCTGCAGTTCTCCACAGGGCTGCCTCCTCAGCCTAAGGACGCAATGACCCTGCCTGCAAGCAGCGTTCCACATATCACTGTGGAGGAGGAAGATGGAGAAATCAGGTTATTGGTCATCCGTGC |
| HU-15043-1 | NOTCH1 | GCTACGAGTGTGCCTGTGAGCCGGGCTACACAGGGAGCATGTGTAACATCAACATCGATGAGTGTGCGGGCAACCCCTGCCACAACGGGGGCACCTGCGAGGACGGCATCAATGGCTTCACCTGCCGCTGCCCCGAGGGCTACCACGACCCCACCTGCCTGTCTGAGGTCAATGAGTGCAACAGCAACCCCTGCGTCCACGGGGCCTGCCGGGACAGCCTCAACGGGTACAAGTGCGACTGTGACCCTGGGTGGAGTGGGACCAACTGTGACATCAACAACAATGAGTGTGAATCCAACCCTTGTGTCAACGGCGGCACCTGCAAAGACATGACCAGTGGCTACGTGTGCACCTGCCGGGAGGGCTTCAGCGGTCCCAACTGCCAGACCAACATCAACGAGTGTGCGTCCAACCCATGTCTGAACCAGGGCACGTGTATTGACGACGTTGCCGGGTACAAGTGCAACTGCCT |
