# Supplementary material for: Whole exome sequencing identifies mTOR and KEAP1 as potential targets for radiosensitization of HNSCC cells refractory to EGFR and β1 integrin inhibition
Source: Oncotarget. 2018 Apr 6;9(26):18099–114. doi: 10.18632/oncotarget.24266 (PMC5915060; doi:10.18632/oncotarget.24266)
Supplement: Supplementary file 3 [file oncotarget-09-18099-s003.docx]

**Supplementary Table 3:** Results of the esiRNA screen in 3D grown FaDu cells for the indicated treatments. P-values are referring to the corresponding RLUC controls.

| **esiRNA** | **Treatment** | **Gy** | **Mean** | **SEM** | **P-value** |
| --- | --- | --- | --- | --- | --- |
| RLUC | IgG | 0 | 1.0000 | 0.0000 |  |
| ERBB3 | IgG | 0 | 1.5031 | 0.1822 | 0.1596 |
| CASP8 | IgG | 0 | 1.3345 | 0.2227 | 0.1214 |
| RAF1 | IgG | 0 | 1.0148 | 0.3087 | 0.9415 |
| KRAS | IgG | 0 | 1.0049 | 0.1727 | 0.9652 |
| GPR98 | IgG | 0 | 0.8898 | 0.3102 | 0.6011 |
| KEAP1 | IgG | 0 | 0.8887 | 0.3793 | 0.6619 |
| P2RY8 | IgG | 0 | 0.8774 | 0.1450 | 0.4436 |
| FRAP1 | IgG | 0 | 0.8745 | 0.3860 | 0.6301 |
| ERBB4 | IgG | 0 | 0.8050 | 0.2880 | 0.3617 |
| **NOTCH1** | IgG | 0 | 0.7610 | 0.0961 | **0.0499** |
| CASC5 | IgG | 0 | 0.7574 | 0.1201 | 0.0729 |
| GPC3 | IgG | 0 | 0.7532 | 0.3436 | 0.3395 |
| POLQ | IgG | 0 | 0.7440 | 0.3125 | 0.2918 |
| FANCD2 | IgG | 0 | 0.7143 | 0.3162 | 0.2581 |
| EP300 | IgG | 0 | 0.6994 | 0.4767 | 0.3888 |
| ETV1 | IgG | 0 | 0.6926 | 0.2693 | 0.1867 |
| PAX3 | IgG | 0 | 0.6821 | 0.2398 | 0.1485 |
| LAMA1 | IgG | 0 | 0.6776 | 0.2138 | 0.1206 |
| MSH2 | IgG | 0 | 0.6373 | 0.2667 | 0.1427 |
| **ARHGEF12** | IgG | 0 | 0.5986 | 0.1178 | **0.0275** |
| RB1 | IgG | 0 | 0.5819 | 0.2011 | 0.0692 |
| **ARID1B** | IgG | 0 | 0.5799 | 0.2577 | 0.1059 |
| **RHOA** | IgG | 0 | 0.5626 | 0.1694 | **0.0465** |
| TRAF7 | IgG | 0 | 0.3685 | 0.0620 | **0.0441** |
| RLUC | AIIB2 | 0 | 1.0000 | 0.0000 |  |
| CASP8 | AIIB2 | 0 | 1.3227 | 0.2632 | 0.1677 |
| KRAS | AIIB2 | 0 | 1.0312 | 0.0433 | 0.3383 |
| CASC5 | AIIB2 | 0 | 0.9577 | 0.1874 | 0.7336 |
| RAF1 | AIIB2 | 0 | 0.8803 | 0.3010 | 0.5623 |
| ETV1 | AIIB2 | 0 | 0.8338 | 0.4226 | 0.5661 |
| ERBB3 | AIIB2 | 0 | 0.7982 | 0.1629 | 0.3301 |
| PAX3 | AIIB2 | 0 | 0.7803 | 0.3528 | 0.3936 |
| FRAP1 | AIIB2 | 0 | 0.7134 | 0.2443 | 0.1793 |
| ARHGEF12 | AIIB2 | 0 | 0.7120 | 0.2363 | 0.3347 |
| ERBB4 | AIIB2 | 0 | 0.6931 | 0.1714 | 0.2394 |
| GPC3 | AIIB2 | 0 | 0.6565 | 0.3975 | 0.2731 |
| MSH2 | AIIB2 | 0 | 0.6462 | 0.3124 | 0.1888 |
| NOTCH1 | AIIB2 | 0 | 0.6445 | 0.1821 | 0.0774 |
| **GPR98** | AIIB2 | 0 | 0.6376 | 0.0589 | **0.0087** |
| **POLQ** | AIIB2 | 0 | 0.6280 | 0.0674 | **0.0108** |
| **KEAP1** | AIIB2 | 0 | 0.6234 | 0.1349 | **0.0402** |
| EP300 | AIIB2 | 0 | 0.6148 | 0.3715 | 0.2144 |
| **RHOA** | AIIB2 | 0 | 0.6076 | 0.0519 | **0.0058** |
| P2RY8 | AIIB2 | 0 | 0.5979 | 0.3095 | 0.3173 |
| **ARID1B** | AIIB2 | 0 | 0.5606 | 0.1680 | **0.0454** |
| **LAMA1** | AIIB2 | 0 | 0.5569 | 0.1472 | **0.0349** |
| FANCD2 | AIIB2 | 0 | 0.5112 | 0.2785 | 0.0933 |
| **RB1** | AIIB2 | 0 | 0.4856 | 0.1535 | **0.0284** |

| **TRAF7** | AIIB2 | 0 | 0.3070 | 0.0744 | **0.0038** |
| --- | --- | --- | --- | --- | --- |
| RLUC | Cetuximab | 0 | 1.0000 | 0.0000 |  |
| CASP8 | Cetuximab | 0 | 0.8119 | 0.1281 | 0.1259 |
| ERBB3 | Cetuximab | 0 | 0.7900 | 0.1477 | 0.1328 |
| KRAS | Cetuximab | 0 | 0.7605 | 0.1772 | 0.1441 |
| RAF1 | Cetuximab | 0 | 0.7031 | 0.0577 | 0.0124 |
| POLQ | Cetuximab | 0 | 0.6266 | 0.2982 | 0.1623 |
| **CASC5** | Cetuximab | 0 | 0.6091 | 0.0546 | **0.0064** |
| FRAP1 | Cetuximab | 0 | 0.6060 | 0.1697 | 0.0567 |
| ERBB4 | Cetuximab | 0 | 0.5718 | 0.1739 | 0.0508 |
| **PAX3** | Cetuximab | 0 | 0.5680 | 0.0665 | **0.0078** |
| **KEAP1** | Cetuximab | 0 | 0.5526 | 0.1200 | **0.0231** |
| MSH2 | Cetuximab | 0 | 0.5206 | 0.2035 | 0.0551 |
| GPR98 | Cetuximab | 0 | 0.5064 | 0.2126 | 0.0567 |
| ARHGEF12 | Cetuximab | 0 | 0.5015 | 0.2587 | 0.0792 |
| **GPC3** | Cetuximab | 0 | 0.4977 | 0.1976 | **0.0479** |
| TRAF7 | Cetuximab | 0 | 0.4697 | 0.2985 | 0.0914 |
| **LAMA1** | Cetuximab | 0 | 0.4547 | 0.1123 | **0.0138** |
| **NOTCH1** | Cetuximab | 0 | 0.4542 | 0.0814 | **0.0073** |
| **ETV1** | Cetuximab | 0 | 0.4450 | 0.0738 | **0.0058** |
| **EP300** | Cetuximab | 0 | 0.4218 | 0.1527 | **0.0225** |
| **FANCD2** | Cetuximab | 0 | 0.3895 | 0.0433 | **0.0017** |
| **ARID1B** | Cetuximab | 0 | 0.3810 | 0.0513 | **0.0023** |
| **P2RY8** | Cetuximab | 0 | 0.3739 | 0.3212 | **0.0777** |
| **RB1** | Cetuximab | 0 | 0.3214 | 0.0204 | **0.0003** |
| **RHOA** | Cetuximab | 0 | 0.3150 | 0.1036 | **0.0075** |
| RLUC | AIIB2 + Cetuximab | 0 | 1.0000 | 0.0000 |  |
| ERBB3 | AIIB2 + Cetuximab | 0 | 1.3477 | 0.1837 | 0.2276 |
| CASP8 | AIIB2 + Cetuximab | 0 | 1.0392 | 0.1681 | 0.7255 |
| RAF1 | AIIB2 + Cetuximab | 0 | 0.8978 | 0.2551 | 0.5596 |
| KRAS | AIIB2 + Cetuximab | 0 | 0.8950 | 0.0840 | 0.1625 |
| KEAP1 | AIIB2 + Cetuximab | 0 | 0.6553 | 0.2063 | 0.1016 |
| POLQ | AIIB2 + Cetuximab | 0 | 0.6351 | 0.1778 | 0.0708 |
| CASC5 | AIIB2 + Cetuximab | 0 | 0.6328 | 0.1786 | 0.0706 |
| FRAP1 | AIIB2 + Cetuximab | 0 | 0.6311 | 0.2195 | 0.1005 |
| ERBB4 | AIIB2 + Cetuximab | 0 | 0.6244 | 0.1690 | 0.0614 |
| **P2RY8** | AIIB2 + Cetuximab | 0 | 0.6110 | 0.0207 | **0.0240** |
| **ARHGEF12** | AIIB2 + Cetuximab | 0 | 0.5475 | 0.0675 | **0.0073** |
| TRAF7 | AIIB2 + Cetuximab | 0 | 0.5176 | 0.2473 | 0.0776 |
| **PAX3** | AIIB2 + Cetuximab | 0 | 0.5081 | 0.1122 | **0.0169** |
| **ETV1** | AIIB2 + Cetuximab | 0 | 0.5064 | 0.1068 | **0.0152** |
| **RB1** | AIIB2 + Cetuximab | 0 | 0.4937 | 0.1393 | **0.0243** |
| **LAMA1** | AIIB2 + Cetuximab | 0 | 0.4859 | 0.1844 | **0.0403** |
| **EP300** | AIIB2 + Cetuximab | 0 | 0.4840 | 0.1292 | **0.0203** |
| **FANCD2** | AIIB2 + Cetuximab | 0 | 0.4782 | 0.1706 | **0.0338** |
| **GPR98** | AIIB2 + Cetuximab | 0 | 0.4729 | 0.0488 | **0.0416** |
| **MSH2** | AIIB2 + Cetuximab | 0 | 0.4623 | 0.1125 | **0.0143** |
| **NOTCH1** | AIIB2 + Cetuximab | 0 | 0.4443 | 0.1198 | **0.0151** |
| **RHOA** | AIIB2 + Cetuximab | 0 | 0.4339 | 0.1446 | **0.0211** |
| GPC3 | AIIB2 + Cetuximab | 0 | 0.4157 | 0.1053 | 0.0807 |
| **ARID1B** | AIIB2 + Cetuximab | 0 | 0.3778 | 0.0369 | **0.0267** |
| RLUC | IgG | 6 | 0.5303 | 0.1450 |  |
| ARID1B | IgG | 6 | 0.6581 | 0.2523 | 0.5201 |

| PAX3 | IgG | 6 | 0.6393 | 0.0942 | 0.3349 |
| --- | --- | --- | --- | --- | --- |
| POLQ | IgG | 6 | 0.5597 | 0.1040 | 0.7198 |
| FRAP1 | IgG | 6 | 0.5538 | 0.1586 | 0.8425 |
| ARHGEF12 | IgG | 6 | 0.5490 | 0.1105 | 0.9108 |
| ETV1 | IgG | 6 | 0.5429 | 0.1023 | 0.8722 |
| NOTCH1 | IgG | 6 | 0.5348 | 0.2361 | 0.9415 |
| RHOA | IgG | 6 | 0.5345 | 0.0850 | 0.9681 |
| ERBB4 | IgG | 6 | 0.5280 | 0.0524 | 0.9832 |
| MSH2 | IgG | 6 | 0.5248 | 0.1000 | 0.9700 |
| RB1 | IgG | 6 | 0.5149 | 0.2384 | 0.8866 |
| TRAF7 | IgG | 6 | 0.5098 | 0.1015 | 0.2412 |
| RAF1 | IgG | 6 | 0.4805 | 0.0687 | 0.6690 |
| FANCD2 | IgG | 6 | 0.4317 | 0.0452 | 0.4612 |
| KRAS | IgG | 6 | 0.4315 | 0.0452 | 0.2389 |
| CASC5 | IgG | 6 | 0.4209 | 0.0678 | 0.3160 |
| CASP8 | IgG | 6 | 0.4041 | 0.0301 | 0.2044 |
| GPR98 | IgG | 6 | 0.3893 | 0.0844 | 0.1536 |
| LAMA1 | IgG | 6 | 0.3881 | 0.0340 | 0.2301 |
| KEAP1 | IgG | 6 | 0.3808 | 0.0949 | 0.3869 |
| P2RY8 | IgG | 6 | 0.3789 | 0.1164 | 0.2212 |
| ERBB3 | IgG | 6 | 0.3257 | 0.0177 | 0.3404 |
| **EP300** | IgG | 6 | 0.2531 | 0.0475 | **0.0473** |
| **GPC3** | IgG | 6 | 0.2307 | 0.0736 | **0.0433** |
| RLUC | AIIB2 | 6 | 0.5223 | 0.1550 |  |
| TRAF7 | AIIB2 | 6 | 0.5452 | 0.1788 | 0.8688 |
| ARID1B | AIIB2 | 6 | 0.4196 | 0.0658 | 0.3046 |
| FANCD2 | AIIB2 | 6 | 0.4172 | 0.0687 | 0.4177 |
| NOTCH1 | AIIB2 | 6 | 0.3966 | 0.1951 | 0.2312 |
| P2RY8 | AIIB2 | 6 | 0.3962 | 0.0980 | 0.6094 |
| MSH2 | AIIB2 | 6 | 0.3634 | 0.2469 | 0.4941 |
| CASP8 | AIIB2 | 6 | 0.3495 | 0.0725 | 0.0590 |
| EP300 | AIIB2 | 6 | 0.3388 | 0.1886 | 0.3384 |
| PAX3 | AIIB2 | 6 | 0.3343 | 0.0246 | 0.1270 |
| **KRAS** | AIIB2 | 6 | 0.3133 | 0.0828 | **0.0290** |
| FRAP1 | AIIB2 | 6 | 0.3118 | 0.0210 | 0.1025 |
| ETV1 | AIIB2 | 6 | 0.2922 | 0.0450 | 0.0666 |
| ARHGEF12 | AIIB2 | 6 | 0.2876 | 0.1644 | 0.2758 |
| ERBB4 | AIIB2 | 6 | 0.2863 | 0.0898 | 0.0573 |
| RHOA | AIIB2 | 6 | 0.2841 | 0.0593 | 0.0549 |
| RB1 | AIIB2 | 6 | 0.2826 | 0.0636 | 0.0972 |
| GPC3 | AIIB2 | 6 | 0.2798 | 0.0935 | 0.1653 |
| ERBB3 | AIIB2 | 6 | 0.2619 | 0.0234 | 0.1029 |
| POLQ | AIIB2 | 6 | 0.2534 | 0.0345 | 0.0691 |
| KEAP1 | AIIB2 | 6 | 0.2345 | 0.0899 | 0.0516 |
| **LAMA1** | AIIB2 | 6 | 0.2258 | 0.0549 | **0.0394** |
| **RAF1** | AIIB2 | 6 | 0.2244 | 0.0472 | **0.0491** |
| CASC5 | AIIB2 | 6 | 0.2221 | 0.0419 | 0.0731 |
| **GPR98** | AIIB2 | 6 | 0.2070 | 0.0256 | **0.0443** |
| RLUC | Cetuximab | 6 | 0.3178 | 0.0205 |  |
| P2RY8 | Cetuximab | 6 | 0.5170 | 0.4214 | 0.9665 |
| TRAF7 | Cetuximab | 6 | 0.3694 | 0.2874 | 0.5738 |
| ARID1B | Cetuximab | 6 | 0.3621 | 0.1212 | 0.3141 |
| ARHGEF12 | Cetuximab | 6 | 0.3476 | 0.1015 | 0.1341 |

| ERBB4 | Cetuximab | 6 | 0.3266 | 0.1652 | 0.3602 |
| --- | --- | --- | --- | --- | --- |
| KRAS | Cetuximab | 6 | 0.3138 | 0.0730 | 0.2027 |
| POLQ | Cetuximab | 6 | 0.3066 | 0.1667 | 0.3380 |
| CASP8 | Cetuximab | 6 | 0.3043 | 0.1527 | 0.2127 |
| RB1 | Cetuximab | 6 | 0.2954 | 0.0672 | 0.1841 |
| FRAP1 | Cetuximab | 6 | 0.2520 | 0.0939 | 0.1772 |
| NOTCH1 | Cetuximab | 6 | 0.2424 | 0.1033 | 0.1771 |
| FANCD2 | Cetuximab | 6 | 0.2406 | 0.0692 | 0.1402 |
| GPR98 | Cetuximab | 6 | 0.2231 | 0.1485 | 0.3135 |
| MSH2 | Cetuximab | 6 | 0.2170 | 0.1535 | 0.1939 |
| **RHOA** | Cetuximab | 6 | 0.2134 | 0.0533 | **0.0285** |
| ETV1 | Cetuximab | 6 | 0.2044 | 0.0193 | 0.0703 |
| EP300 | Cetuximab | 6 | 0.2041 | 0.0541 | 0.0936 |
| CASC5 | Cetuximab | 6 | 0.2034 | 0.0887 | 0.1249 |
| ERBB3 | Cetuximab | 6 | 0.2021 | 0.1175 | 0.1598 |
| GPC3 | Cetuximab | 6 | 0.2018 | 0.0794 | 0.0990 |
| LAMA1 | Cetuximab | 6 | 0.1973 | 0.0815 | 0.1244 |
| PAX3 | Cetuximab | 6 | 0.1920 | 0.0076 | 0.0514 |
| RAF1 | Cetuximab | 6 | 0.1913 | 0.0150 | 0.0669 |
| **KEAP1** | Cetuximab | 6 | 0.1835 | 0.0126 | **0.0458** |
| RLUC | AIIB2 + Cetuximab | 6 | 0.2295 | 0.0827 |  |
| **NOTCH1** | AIIB2 + Cetuximab | 6 | 0.2346 | 0.0786 | **0.0168** |
| **CASC5** | AIIB2 + Cetuximab | 6 | 0.2284 | 0.0616 | **0.0354** |
| **KRAS** | AIIB2 + Cetuximab | 6 | 0.2257 | 0.0513 | **0.0324** |
| ERBB4 | AIIB2 + Cetuximab | 6 | 0.1939 | 0.0832 | 0.1201 |
| CASP8 | AIIB2 + Cetuximab | 6 | 0.1857 | 0.0303 | 0.0671 |
| ARHGEF12 | AIIB2 + Cetuximab | 6 | 0.1806 | 0.0366 | 0.0655 |
| MSH2 | AIIB2 + Cetuximab | 6 | 0.1711 | 0.0003 | 0.1097 |
| EP300 | AIIB2 + Cetuximab | 6 | 0.1679 | 0.0607 | 0.2908 |
| **PAX3** | AIIB2 + Cetuximab | 6 | 0.1491 | 0.0681 | **0.0476** |
| **ETV1** | AIIB2 + Cetuximab | 6 | 0.1443 | 0.0299 | **0.0376** |
| **ARID1B** | AIIB2 + Cetuximab | 6 | 0.1381 | 0.0296 | **0.0443** |
| **POLQ** | AIIB2 + Cetuximab | 6 | 0.1350 | 0.0278 | **0.0446** |
| **ERBB3** | AIIB2 + Cetuximab | 6 | 0.1344 | 0.0105 | **0.0366** |
| RB1 | AIIB2 + Cetuximab | 6 | 0.1320 | 0.0515 | 0.0718 |
| **GPC3** | AIIB2 + Cetuximab | 6 | 0.1301 | 0.0368 | **0.0491** |
| FANCD2 | AIIB2 + Cetuximab | 6 | 0.1298 | 0.0767 | 0.0849 |
| **LAMA1** | AIIB2 + Cetuximab | 6 | 0.1273 | 0.0135 | **0.0358** |
| **FRAP1** | AIIB2 + Cetuximab | 6 | 0.1251 | 0.0386 | **0.0300** |
| GPR98 | AIIB2 + Cetuximab | 6 | 0.1248 | 0.0157 | 0.1150 |
| **RAF1** | AIIB2 + Cetuximab | 6 | 0.1179 | 0.0318 | **0.0291** |
| P2RY8 | AIIB2 + Cetuximab | 6 | 0.1131 | 0.0349 | 0.2350 |
| **TRAF7** | AIIB2 + Cetuximab | 6 | 0.1011 | 0.0336 | **0.0481** |
| **RHOA** | AIIB2 + Cetuximab | 6 | 0.0797 | 0.0554 | **0.0380** |
| **KEAP1** | AIIB2 + Cetuximab | 6 | 0.0784 | 0.0320 | **0.0446** |
