# Supplementary material for: Whole exome sequencing identifies mTOR and KEAP1 as potential targets for radiosensitization of HNSCC cells refractory to EGFR and β1 integrin inhibition
Source: Oncotarget. 2018 Apr 6;9(26):18099–114. doi: 10.18632/oncotarget.24266 (PMC5915060; doi:10.18632/oncotarget.24266)
Supplement: Supplementary file 4 [file oncotarget-09-18099-s004.docx]

**Supplementary Table 4:** Results of the esiRNA screen in 3D grown SAS cells for the indicated treatments. P-values are referring to the corresponding RLUC controls.

| **esiRNA** | **treatment** | **Gy** | **mean** | **SEM** | **P-value** |
| --- | --- | --- | --- | --- | --- |
| RLUC | IgG | 0 | 1.0000 | 0.0000 |  |
| KRAS | IgG | 0 | 0.9850 | 0.1940 | 0.9059 |
| FANCD2 | IgG | 0 | 0.8523 | 0.0937 | 0.1121 |
| RAF1 | IgG | 0 | 0.7809 | 0.2404 | 0.2551 |
| RB1 | IgG | 0 | 0.7441 | 0.2201 | 0.3479 |
| CASP8 | IgG | 0 | 0.7388 | 0.1775 | 0.1256 |
| FRAP1 | IgG | 0 | 0.7324 | 0.1875 | 0.1320 |
| MSH2 | IgG | 0 | 0.6250 | 0.0752 | 0.0896 |
| **TRAF7** | IgG | 0 | 0.6246 | 0.0845 | **0.0165** |
| **ARHGEF12** | IgG | 0 | 0.6245 | 0.0792 | **0.0145** |
| **KEAP1** | IgG | 0 | 0.6239 | 0.0988 | **0.0222** |
| **ERBB3** | IgG | 0 | 0.5903 | 0.0938 | **0.0170** |
| POLQ | IgG | 0 | 0.5782 | 0.1845 | 0.0583 |
| **P2RY8** | IgG | 0 | 0.5060 | 0.0404 | **0.0022** |
| LAMA1 | IgG | 0 | 0.4946 | 0.1476 | 0.1296 |
| **PAX3** | IgG | 0 | 0.4485 | 0.1723 | **0.0310** |
| EP300 | IgG | 0 | 0.4433 | 0.0821 | 0.0662 |
| **NOTCH1** | IgG | 0 | 0.4317 | 0.1601 | **0.0254** |
| GPC3 | IgG | 0 | 0.4036 | 0.1755 | 0.1306 |
| **GPR98** | IgG | 0 | 0.4027 | 0.0530 | **0.0026** |
| **ARID1B** | IgG | 0 | 0.3828 | 0.0866 | **0.0065** |
| ETV1 | IgG | 0 | 0.3576 | 0.0726 | 0.0508 |
| **RHOA** | IgG | 0 | 0.3472 | 0.0986 | **0.0075** |
| **CASC5** | IgG | 0 | 0.2444 | 0.0024 | **0.0014** |
| ERBB4 | IgG | 0 | 0.2033 | 0.1150 | 0.0648 |
| RLUC | AIIB2 | 0 | 1.0000 | 0.0000 |  |
| KEAP1 | AIIB2 | 0 | 0.8187 | 0.2058 | 0.2665 |
| KRAS | AIIB2 | 0 | 0.7877 | 0.1560 | 0.1425 |
| **ARHGEF12** | AIIB2 | 0 | 0.7848 | 0.0692 | **0.0328** |
| TRAF7 | AIIB2 | 0 | 0.7655 | 0.1594 | 0.2852 |
| RAF1 | AIIB2 | 0 | 0.7434 | 0.2915 | 0.2668 |
| RB1 | AIIB2 | 0 | 0.7325 | 0.2341 | 0.1864 |
| FANCD2 | AIIB2 | 0 | 0.6968 | 0.1808 | 0.1010 |
| ERBB3 | AIIB2 | 0 | 0.6031 | 0.2067 | 0.0797 |
| **CASP8** | AIIB2 | 0 | 0.5970 | 0.1348 | **0.0353** |
| LAMA1 | AIIB2 | 0 | 0.5827 | 0.1941 | 0.0651 |
| ARID1B | AIIB2 | 0 | 0.5761 | 0.2098 | 0.2144 |
| **GPR98** | AIIB2 | 0 | 0.5007 | 0.1763 | **0.0391** |
| **FRAP1** | AIIB2 | 0 | 0.4915 | 0.2033 | **0.0494** |
| P2RY8 | AIIB2 | 0 | 0.4848 | 0.2073 | 0.0500 |
| **POLQ** | AIIB2 | 0 | 0.4837 | 0.1288 | **0.0201** |
| **NOTCH1** | AIIB2 | 0 | 0.4687 | 0.0859 | **0.0086** |
| **MSH2** | AIIB2 | 0 | 0.4053 | 0.1474 | **0.0199** |
| GPC3 | AIIB2 | 0 | 0.3957 | 0.2676 | 0.1932 |
| RHOA | AIIB2 | 0 | 0.3703 | 0.1181 | 0.0839 |
| **ETV1** | AIIB2 | 0 | 0.3560 | 0.0599 | **0.0029** |
| **EP300** | AIIB2 | 0 | 0.3258 | 0.1192 | **0.0103** |
| **CASC5** | AIIB2 | 0 | 0.2824 | 0.0870 | **0.0049** |
| **PAX3** | AIIB2 | 0 | 0.2648 | 0.0364 | **0.0223** |

| **ERBB4** | AIIB2 | 0 | 0.1254 | 0.0419 | **0.0008** |
| --- | --- | --- | --- | --- | --- |
| RLUC | Cetuximab | 0 | 1.0000 | 0.0000 |  |
| RAF1 | Cetuximab | 0 | 0.8583 | 0.2709 | 0.4607 |
| KRAS | Cetuximab | 0 | 0.8021 | 0.1391 | 0.1326 |
| ERBB4 | Cetuximab | 0 | 0.7532 | 0.3682 | 0.3655 |
| KEAP1 | Cetuximab | 0 | 0.7476 | 0.1790 | 0.1346 |
| FANCD2 | Cetuximab | 0 | 0.7307 | 0.1540 | 0.0940 |
| **ERBB3** | Cetuximab | 0 | 0.6303 | 0.1230 | **0.0350** |
| **ARHGEF12** | Cetuximab | 0 | 0.6255 | 0.0948 | **0.0207** |
| CASP8 | Cetuximab | 0 | 0.5751 | 0.2110 | 0.0733 |
| GPC3 | Cetuximab | 0 | 0.5503 | 0.2594 | 0.0953 |
| RB1 | Cetuximab | 0 | 0.5393 | 0.1293 | 0.1247 |
| RHOA | Cetuximab | 0 | 0.5247 | 0.2041 | 0.0563 |
| **FRAP1** | Cetuximab | 0 | 0.4749 | 0.0857 | **0.0088** |
| **TRAF7** | Cetuximab | 0 | 0.4599 | 0.0844 | **0.0080** |
| **LAMA1** | Cetuximab | 0 | 0.4534 | 0.1458 | **0.0229** |
| **GPR98** | Cetuximab | 0 | 0.4361 | 0.0836 | **0.0072** |
| NOTCH1 | Cetuximab | 0 | 0.4194 | 0.2466 | 0.1857 |
| POLQ | Cetuximab | 0 | 0.4014 | 0.1245 | 0.0141 |
| ARID1B | Cetuximab | 0 | 0.3927 | 0.2165 | 0.1572 |
| **MSH2** | Cetuximab | 0 | 0.3571 | 0.0425 | **0.0015** |
| **EP300** | Cetuximab | 0 | 0.3543 | 0.0628 | **0.0437** |
| **PAX3** | Cetuximab | 0 | 0.3249 | 0.0136 | **0.0001** |
| **P2RY8** | Cetuximab | 0 | 0.3209 | 0.0507 | **0.0336** |
| **ETV1** | Cetuximab | 0 | 0.2665 | 0.1265 | **0.0098** |
| **CASC5** | Cetuximab | 0 | 0.2206 | 0.0709 | **0.0027** |
| RLUC | AIIB2 + Cetuximab | 0 | 1.0000 | 0.0000 |  |
| KEAP1 | AIIB2 + Cetuximab | 0 | 0.9990 | 0.0462 | 0.9800 |
| CASP8 | AIIB2 + Cetuximab | 0 | 0.9346 | 0.0305 | 0.2031 |
| **FANCD2** | AIIB2 + Cetuximab | 0 | 0.6828 | 0.0984 | **0.0306** |
| RAF1 | AIIB2 + Cetuximab | 0 | 0.6789 | 0.2361 | 0.3052 |
| RB1 | AIIB2 + Cetuximab | 0 | 0.6551 | 0.2748 | 0.1618 |
| KRAS | AIIB2 + Cetuximab | 0 | 0.6315 | 0.1068 | 0.1287 |
| ERBB3 | AIIB2 + Cetuximab | 0 | 0.6165 | 0.1988 | 0.0791 |
| LAMA1 | AIIB2 + Cetuximab | 0 | 0.5943 | 0.0690 | 0.0762 |
| ARHGEF12 | AIIB2 + Cetuximab | 0 | 0.5792 | 0.0951 | 0.1009 |
| **NOTCH1** | AIIB2 + Cetuximab | 0 | 0.5571 | 0.0450 | **0.0457** |
| TRAF7 | AIIB2 + Cetuximab | 0 | 0.5506 | 0.2834 | 0.2670 |
| **RHOA** | AIIB2 + Cetuximab | 0 | 0.5337 | 0.0388 | **0.0023** |
| ARID1B | AIIB2 + Cetuximab | 0 | 0.4999 | 0.2880 | 0.2462 |
| POLQ | AIIB2 + Cetuximab | 0 | 0.4841 | 0.2105 | 0.0513 |
| MSH2 | AIIB2 + Cetuximab | 0 | 0.4802 | 0.2935 | 0.2418 |
| **GPR98** | AIIB2 + Cetuximab | 0 | 0.4250 | 0.0085 | **0.0066** |
| GPC3 | AIIB2 + Cetuximab | 0 | 0.4198 | 0.0795 | 0.0615 |
| PAX3 | AIIB2 + Cetuximab | 0 | 0.3616 | 0.2688 | 0.0543 |
| ERBB4 | AIIB2 + Cetuximab | 0 | 0.3241 | 0.0511 | **0.0340** |
| **EP300** | AIIB2 + Cetuximab | 0 | 0.3164 | 0.0617 | **0.0406** |
| **ETV1** | AIIB2 + Cetuximab | 0 | 0.2962 | 0.0618 | **0.0395** |
| **P2RY8** | AIIB2 + Cetuximab | 0 | 0.2851 | 0.0016 | **0.0010** |
| **FRAP1** | AIIB2 + Cetuximab | 0 | 0.2551 | 0.0465 | **0.0281** |
| **CASC5** | AIIB2 + Cetuximab | 0 | 0.2360 | 0.1374 | **0.0106** |
| RLUC | IgG | 6 | 0.7694 | 0.0876 |  |
| **CASC5** | IgG | 6 | 1.1094 | 0.1547 | **0.0473** |

| ERBB4 | IgG | 6 | 1.0793 | 0.2650 | 0.4236 |
| --- | --- | --- | --- | --- | --- |
| RB1 | IgG | 6 | 0.8250 | 0.0635 | 0.5700 |
| EP300 | IgG | 6 | 0.8056 | 0.0231 | 0.5543 |
| GPR98 | IgG | 6 | 0.7681 | 0.0321 | 0.9782 |
| RHOA | IgG | 6 | 0.7355 | 0.1493 | 0.7787 |
| NOTCH1 | IgG | 6 | 0.7346 | 0.0630 | 0.5854 |
| KRAS | IgG | 6 | 0.7228 | 0.0461 | 0.5251 |
| PAX3 | IgG | 6 | 0.7193 | 0.1826 | 0.4589 |
| ARID1B | IgG | 6 | 0.7044 | 0.0858 | 0.5664 |
| GPC3 | IgG | 6 | 0.6833 | 0.1885 | 0.3982 |
| ARHGEF12 | IgG | 6 | 0.6645 | 0.0611 | 0.2344 |
| KEAP1 | IgG | 6 | 0.6271 | 0.1347 | 0.1022 |
| CASP8 | IgG | 6 | 0.6140 | 0.1759 | 0.2753 |
| P2RY8 | IgG | 6 | 0.6123 | 0.1651 | 0.3873 |
| MSH2 | IgG | 6 | 0.6052 | 0.1294 | 0.1226 |
| **TRAF7** | IgG | 6 | 0.6030 | 0.0748 | **0.0318** |
| RAF1 | IgG | 6 | 0.6022 | 0.1926 | 0.3521 |
| LAMA1 | IgG | 6 | 0.5972 | 0.0098 | 0.2559 |
| ETV1 | IgG | 6 | 0.5950 | 0.1531 | 0.5664 |
| ERBB3 | IgG | 6 | 0.5695 | 0.1136 | 0.2216 |
| FANCD2 | IgG | 6 | 0.5687 | 0.1350 | 0.1255 |
| **POLQ** | IgG | 6 | 0.5676 | 0.0964 | **0.0093** |
| **FRAP1** | IgG | 6 | 0.4070 | 0.0577 | **0.0391** |
| RLUC | AIIB2 | 6 | 0.6236 | 0.0637 |  |
| RHOA | AIIB2 | 6 | 0.9622 | 0.2768 | 0.5900 |
| ERBB4 | AIIB2 | 6 | 0.9612 | 0.1385 | 0.1335 |
| MSH2 | AIIB2 | 6 | 0.8967 | 0.2162 | 0.5420 |
| P2RY8 | AIIB2 | 6 | 0.8690 | 0.1144 | 0.0569 |
| GPC3 | AIIB2 | 6 | 0.8289 | 0.2234 | 0.4967 |
| RAF1 | AIIB2 | 6 | 0.7174 | 0.0473 | 0.3555 |
| KRAS | AIIB2 | 6 | 0.6958 | 0.1470 | 0.3758 |
| CASP8 | AIIB2 | 6 | 0.6930 | 0.0985 | 0.2149 |
| ERBB3 | AIIB2 | 6 | 0.6891 | 0.0836 | 0.0838 |
| POLQ | AIIB2 | 6 | 0.6756 | 0.1721 | 0.2426 |
| FRAP1 | AIIB2 | 6 | 0.6688 | 0.0596 | 0.3452 |
| FANCD2 | AIIB2 | 6 | 0.6587 | 0.1399 | 0.0715 |
| NOTCH1 | AIIB2 | 6 | 0.6488 | 0.1681 | 0.4903 |
| PAX3 | AIIB2 | 6 | 0.6226 | 0.1641 | 0.1587 |
| **ARHGEF12** | AIIB2 | 6 | 0.5999 | 0.0999 | **0.0156** |
| CASC5 | AIIB2 | 6 | 0.5807 | 0.1048 | 0.0870 |
| **LAMA1** | AIIB2 | 6 | 0.5449 | 0.0640 | **0.0040** |
| RB1 | AIIB2 | 6 | 0.5378 | 0.1285 | 0.1405 |
| ETV1 | AIIB2 | 6 | 0.5336 | 0.1806 | 0.2576 |
| **EP300** | AIIB2 | 6 | 0.5262 | 0.0572 | **0.0072** |
| ARID1B | AIIB2 | 6 | 0.5083 | 0.0914 | 0.3313 |
| GPR98 | AIIB2 | 6 | 0.5080 | 0.1085 | 0.1201 |
| **KEAP1** | AIIB2 | 6 | 0.4717 | 0.0052 | **0.0274** |
| TRAF7 | AIIB2 | 6 | 0.4115 | 0.0802 | 0.2300 |
| RLUC | Cetuximab | 6 | 0.7844 | 0.0905 |  |
| POLQ | Cetuximab | 6 | 1.1546 | 0.2372 | 0.1212 |
| ARID1B | Cetuximab | 6 | 1.0926 | 0.2078 | 0.3788 |
| ETV1 | Cetuximab | 6 | 0.9900 | 0.1492 | 0.1079 |
| CASC5 | Cetuximab | 6 | 0.9230 | 0.0537 | 0.1945 |

| GPC3 | Cetuximab | 6 | 0.8827 | 0.1792 | 0.3496 |
| --- | --- | --- | --- | --- | --- |
| NOTCH1 | Cetuximab | 6 | 0.8187 | 0.2275 | 0.9990 |
| KRAS | Cetuximab | 6 | 0.7958 | 0.1305 | 0.8467 |
| RB1 | Cetuximab | 6 | 0.7821 | 0.1030 | 0.8825 |
| LAMA1 | Cetuximab | 6 | 0.7144 | 0.0543 | 0.3393 |
| ERBB3 | Cetuximab | 6 | 0.7099 | 0.0963 | 0.5864 |
| MSH2 | Cetuximab | 6 | 0.7065 | 0.1416 | 0.6487 |
| ARHGEF12 | Cetuximab | 6 | 0.6536 | 0.1655 | 0.4445 |
| CASP8 | Cetuximab | 6 | 0.6441 | 0.0862 | 0.0856 |
| TRAF7 | Cetuximab | 6 | 0.6292 | 0.2007 | 0.4387 |
| RAF1 | Cetuximab | 6 | 0.6226 | 0.0810 | 0.2464 |
| RHOA | Cetuximab | 6 | 0.6208 | 0.1791 | 0.3598 |
| P2RY8 | Cetuximab | 6 | 0.6004 | 0.0800 | 0.1143 |
| PAX3 | Cetuximab | 6 | 0.5969 | 0.1291 | 0.2090 |
| FANCD2 | Cetuximab | 6 | 0.5938 | 0.0994 | 0.2320 |
| EP300 | Cetuximab | 6 | 0.5899 | 0.0808 | 0.0996 |
| GPR98 | Cetuximab | 6 | 0.5697 | 0.0089 | 0.2882 |
| KEAP1 | Cetuximab | 6 | 0.5453 | 0.1036 | 0.1727 |
| FRAP1 | Cetuximab | 6 | 0.5323 | 0.1038 | 0.1640 |
| **ERBB4** | Cetuximab | 6 | 0.2653 | 0.0353 | **0.0070** |
| RLUC | AIIB2 + Cetuximab | 6 | 0.6987 | 0.0367 |  |
| RAF1 | AIIB2 + Cetuximab | 6 | 1.3513 | 0.2324 | 0.2281 |
| P2RY8 | AIIB2 + Cetuximab | 6 | 1.1854 | 0.2955 | 0.3779 |
| CASC5 | AIIB2 + Cetuximab | 6 | 1.0293 | 0.2815 | 0.3460 |
| ERBB4 | AIIB2 + Cetuximab | 6 | 1.0056 | 0.2829 | 0.2766 |
| FRAP1 | AIIB2 + Cetuximab | 6 | 0.9381 | 0.1268 | 0.4776 |
| KRAS | AIIB2 + Cetuximab | 6 | 0.8081 | 0.1595 | 0.8255 |
| FANCD2 | AIIB2 + Cetuximab | 6 | 0.7706 | 0.0767 | 0.9915 |
| TRAF7 | AIIB2 + Cetuximab | 6 | 0.7697 | 0.0279 | 0.4066 |
| ERBB3 | AIIB2 + Cetuximab | 6 | 0.7439 | 0.1937 | 0.8891 |
| GPC3 | AIIB2 + Cetuximab | 6 | 0.7166 | 0.0865 | 0.3421 |
| ETV1 | AIIB2 + Cetuximab | 6 | 0.7122 | 0.0864 | 0.2499 |
| RB1 | AIIB2 + Cetuximab | 6 | 0.6767 | 0.0731 | 0.3989 |
| GPR98 | AIIB2 + Cetuximab | 6 | 0.6555 | 0.1536 | 0.4167 |
| EP300 | AIIB2 + Cetuximab | 6 | 0.6395 | 0.1013 | 0.5965 |
| RHOA | AIIB2 + Cetuximab | 6 | 0.6377 | 0.1659 | 0.1595 |
| POLQ | AIIB2 + Cetuximab | 6 | 0.6365 | 0.0844 | 0.1646 |
| PAX3 | AIIB2 + Cetuximab | 6 | 0.6170 | 0.3049 | 0.5230 |
| MSH2 | AIIB2 + Cetuximab | 6 | 0.6070 | 0.1251 | 0.2934 |
| ARHGEF12 | AIIB2 + Cetuximab | 6 | 0.6016 | 0.0994 | 0.5035 |
| NOTCH1 | AIIB2 + Cetuximab | 6 | 0.5437 | 0.2374 | 0.3193 |
| CASP8 | AIIB2 + Cetuximab | 6 | 0.5252 | 0.0938 | 0.1062 |
| ARID1B | AIIB2 + Cetuximab | 6 | 0.5108 | 0.1278 | 0.2131 |
| **LAMA1** | AIIB2 + Cetuximab | 6 | 0.4474 | 0.0485 | **0.0299** |
| **KEAP1** | AIIB2 + Cetuximab | 6 | 0.3982 | 0.0430 | **0.0204** |
